# Efflux pumps as an additional source of resistance to trichothecenes in *Fusarium proliferatum* and *Fusarium oxysporum* isolates

**DOI:** 10.1007/s13353-019-00501-2

**Published:** 2019-06-27

**Authors:** Delfina Popiel, Adam Dawidziuk, Grzegorz Koczyk

**Affiliations:** 1 Department of Pathogen Genetics and Plant Resistance, Institute of Plant Genetics, Polish Academy of Sciences, Strzeszynska 34, Poznan, Poland; 2 Department of Biometry and Bioinformatics, Institute of Plant Genetics, Polish Academy of Sciences, Strzeszynska 34, Poznan, Poland

**Keywords:** Trichothecene efflux pump, *Fusarium oxysporum*, *Tri12*, Transcription factor binding site

## Abstract

Role of efflux-mediated toxin resistance to trichothecenes is known in trichothecene-producing species. However, the role of trichothecene efflux pump homologues in non-producing fusaria such as *F. oxysporum* and *F. proliferatum* was not investigated in detail. Analysis of the homologues of trichothecene efflux pump from multiple fungal species allowed us to uncover and catalogue functional gene copies of conserved structure. Putative Tri12 candidates in *Fusarium oxysporum* and *F. proliferatum* were characterised via expression profiling in response to different trigger compounds, providing supporting evidence for role of *Tri12* homologues in the resistance to trichothecenes. Our analysis of *Tri12* phylogeny also suggests that efflux-mediated trichothecene resistance is likely to predate the divergence of *Trichoderma* and *Fusarium* species. On the regulatory level, we posit that the increased tolerance of trichothecenes by *F. oxysporum* is possibly related to the decoupling of *Tri12* homologue expression from pH, due to the deletion of *PACC/RIM101* transcription factor binding site in its promoter region.

**Electronic supplementary material:**

The online version of this article (10.1007/s13353-019-00501-2) contains supplementary material, which is available to authorized users.

## Introduction

Mycotoxins are bioactive fungal secondary metabolites which are typically viewed mainly through the lens of their harmful properties to humans and livestock. Nevertheless, a large array of fungal pathogens spend most of their life cycle, in competition with prokaryotic and eukaryotic microbes (either persisting in the soil environment or competing for plant hosts). Thus, one can expect that many compounds affect fungal fitness through their effect on the microbe-microbe competition. In cases of some toxins, the divergence and spread of different toxigenic properties have been shown to predate the origins of major classes of filamentous fungi (e.g. Koczyk et al. 2015), with detoxification mechanisms likewise being ancient (e.g. Popiel et al. 2014; Perlin et al. 2014). In the different environments, the competitive impact of toxin biosynthesis can be positive, due to increased isolate virulence or toxin’s inhibitory effects on the growth of other strains (Losada et al. 2009).

In this context, there is evidence demonstrating that trichothecene biosynthesis has impact on fungus-fungus competition. For example, Ramakrishna et al. (1996) found that during competition between *F. sporotrichioides* (producer of T-2 toxin) and two other fungi: *A. flavus* and *Penicillium verrucosum* the growth of *F. sporotrichioides* was negatively affected but paradoxically the production of T-2 mycotoxin was stimulated. Similarly, Lutz et al. (2003) tested the impact of deoxynivalenol (DON) against a potent fungal antagonist *Trichoderma atroviride* and described decreased expression of genes coding chitinase in the presence of these toxins. In the studies of McLaughlin et al. (2009) and Bin-Umer et al. (2011), they observed the impact of trichothecenes on yeast cells. The results illustrated that mycotoxins can inhibit the mitochondrial membrane potential, translation and levels of reactive oxygen species in fungi, in a dose-dependent manner.

Trichothecene biosynthesis contributes to increased virulence of fungal strains, and further inquiries into other fungal species show that the basic trichothecene scaffold is likely ancient at least within the context of multiple species within the *Hypocreales* order (*Trichoderma arundinaceum* and *Trichoderma brevicompactum—*Cardoza et al. 2011; *Myrothecium roridum—*Trapp et al. 1998; *Stachybotrys* sp.*—*Semeiks et al. 2014)*.* While capacity for trichothecene biosynthesis is present in multiple members of the *Hypocreales* order, the *Fusarium* genus is perhaps the best characterised group (Kimura et al. 2007). *Fusarium* spp. are subdivided into related, but phylogenetically distinct complexes that likely diverged in cretaceous period (O’Donnell et al. 2013). These fungi widely differ in their preferences in regards to saprophytic and/or pathogenic lifestyles as well as biosynthetic capabilities (trichothecenes are mainly produced by members of *incarnatum-equiseti* and *sambucinum* complexes). Frequently, the diverged species find themselves in direct or indirect competition when their ecological niches overlap. The relationship between *F. graminearum* and *F. verticillioides* on cereals (including variability in trichothecene accumulation) (Picot et al. 2012; Dawidziuk et al. 2016) provides one example of this phenomenon.

Mirroring the ancient origins of trichothecene biosynthesis, effective trichothecene resistance mechanisms are known to be partially present in multiple producing and non-producing strains (Kimura et al. 2003; Tokai et al. 2005; Proctor et al. 2009; Menke et al. 2012). A good example is trichotecene effux pump encoded by *Tri12* geneor O-acetyltransferase Tri101 (and its divergent but functional Tri201 homologue). The *Tri201* gene, in particular, was found to be present in both ancestrally divergent strains of *Fusarium* sp. from complexes other than *sambucinum*, e.g. the early diverging species *F. decemcellulare*, *F. solani*(Tokai et al. 2005) as well as in other species of fungi (*Magnaporthe oryza—*Tokai et al. 2005; *Saccharomyces cerevisiae—*Alexander et al. 2002).

While the trichothecene acetyltransferase homologues have been characterised in many species, the putative trichothecene efflux pump existence and functionality were not extensively investigated beyond the initial discovery of their functionality in the *sambucinum* complex (*F. sporotrichioides—*Alexander et al. 1999; *F. graminearum—*Wuchiyama et al. 2000). In producer species, the past comprehensive studies of Proctor and coworkers (Proctor et al. 2009; Cardoza et al. 2011) have shown that trichothecene efflux pump is frequently but not always present in the cluster (e.g. *incarnatum-equiseti* complex fusaria). More recently, a brief survey of *Tri12* domain encoding transporters was conducted by Perlin et al. (2014), where homologues of unconfirmed function were summarised across many saprobic, animal and plant pathogenic-species.

To establish whether trichothecene efflux is a likely retained trait in previously not investigated species (*F. oxysporum*, *F. proliferatum*), we studied presence and evolutionary history of divergent *Tri12* homologues. The research was performed by combining phylogenetic analyses of available *Ascomycota* sequences with gene expression and bioassays. Through the phylogenetic analysis of multiple *Tri12* homologues, we confirmed the notion that active resistance to trichothecene-like compounds is likely an ancient trait or one common enough to elicit a convergent evolution of multiple, distantly related resistance factors (acetyltransferase and active transport). In order to obtain supporting evidence for functionality of divergent Tri12 homologues in *fujikuroi* and *oxysporum* complex, we investigated the expression of *F. proliferatum* and *F. oxysporum* homologues in response to varying stimuli (including trichothecene presence).

## Methods

### Isolate collection and identification

From the culture collections available at the Institute of Plant Genetics, Polish Academy of Sciences, Poznan, Poland, we selected the following strains lacking capacity for trichothecene biosynthesis: eight *F. oxysporum* strains (10 L, 11 L, 19 L, 55 L, 57 L, 94 L, 115 L, 131 L) with no recorded toxigenic potential (fumonisins, trichothecenes, zearalenone) and ten *F. proliferatum* strains (1 L, 3 L, 7 L, 21 L, 36 L, 58 L, 59 L, 66 L, 81 L, 99 L) known to produce fumonisins. Fungal strain annotation was conducted as per the protocol described in Dawidziuk et al. (2014) on basis of both morphological and molecular data: *F. oxysporum* strains 10 L—GenBank Accession number MN018756, 11 L-MN018757, 19 L-KF889103, 55 L-KF889104, 57 L-KF889105, 94 L-MN018758, 115 L-KF889099, 131 L-KF889101 and *F. proliferatum* strains 1 L-KF889131, 3 L-KF889134, 7 L-KF889137, 21 L-KF889122, 36 L-MN018759, 58 L-KF889136, 59 L-KF889132, 66 L-KF889125, 81 L-MN018760, 99 L-KF889127).

### Bioassays with multiple compounds

For the purpose of the bioassay experiment, the concentration of deoxynivalenol was set to 8 mg/L as the lowest dose inhibiting fungal growth. The lower concentrations of toxin (1 mg/L, 2 mg/L and 5 mg/L) did not significantly influence the growth of the isolates and the higher doses (10 mg/L) inhibited growth of all fungal cultures.

The additional bioassay experiments were carried out to eliminate the impact of environmental factors on the growth of the tested cultures: MgCl_2_, KCl, ferulic acid, fungicide-Alert 350 SC (flusilazole) ground wheat seedlings, glucose, sucrose, coumaric acid, H_2_O_2_, caffeine, *F. verticillioides* (fumonisin producer). The concentration of additional chemical compounds was set to the same value as the concentration of DON. In the case of additional biological compounds, the 5 g of ground wheat was added to 250 mL of PDA medium and *F. oxysporum*/*F. proliferatum* bioassays with *F. verticillioides* was tested in the dual cultures (Gromadzka et al. 2009). The response was observed on PDA medium amended in simulated day (16 h)/night (8 h) conditions at 25 °C. The *F. oxysporum* assay was performed in three biological and ten technical replicates and the *F. proliferatum* assay was performed in three biological and ten technical replicates. Biological replicates were performed separately in the phytotron strictly controlling temperature, humidity and simulated day/night conditions.

The surface area of the fungal colonies was calculated by approximating the mycelium’s area to an ellipse by measuring both the length and width of the mycelium 4 days after toxin exposition (Dawidziuk et al. 2016).

### Isolation of DNA and sequencing

Mycelium used for DNA extraction was obtained by inoculating Czapek-Dox broth (Sigma Aldrich, St. Louis, Missouri, USA) with yeast extract (Oxoid, Waltham, Massachusetts, USA) and streptomycin sulphate (50 mg/L, AppliChem, Darmstadt, Germany) and after incubation at 25 °C on a rotary shaker (120 rpm). Mycelium was collected on filter paper in a Büchner funnel, washed with sterile water, frozen at − 20 °C and freeze-dried. Total DNA was extracted using the DNeasy Plant Mini Kit (Qiagen, Hilden, Germany). The quality of DNA was estimated by a NanoDrop 2000 UV-Vis Spectrophotometer (Thermo Scientific, Wilmington, USA) and a Experion Automated Electrophoresis System (Bio-Rad, Hercules, CA). The protocols for primer design, PCR and sequencing conditions have been previously described by Popiel et al. (2014) and sequences of the primers are listed in Table 1.

**Table 1:** The sequences of the primers used for amplification and gene expression

Gene targeted	Primer name	Primer sequence (5′ to 3′)	Protocol
Trichothecene efflux pump (*Tri12*)	preTRI12_F1	ACGGAAGATCCGAGAGCTTCA	Amplification and sequencing
	preTRI12_R1	GCCGATGTGCTGGTTGATGTT	
	preTRI12_F2	ACGAGCTCAGTACGAGGTACA	
	preTRI12_R2	GCGGGACGCTATAATGATCGA	
	preTRI12_F3	TTGGACAAGTCGGTGACGGAA	
	preTRI12_R3	GTTGAGAGACCGTCCACACAA	
	preTRI12_F4	TCCTCGTGACCGATAGATACG	
	preTRI12_R4	GTCATTGTGACCCGGAGAGAT	
	TRI12_F1	CCCTCTGGTACTTCTTCTACC	
	TRI12_R1	GACTTTGGCGTTGATGACACG	
	TRI12_F2	CTTGCAGCGACATACTTTGCC	
	TRI12_R2	GGCCCGAGACAAGAAGAAAAG	
	TRI12_F3	GGATGTCCGAGGTTACACATG	
	TRI12_R3	CACGGTGCTCAATATGCTTCG	
	TRI12_F4	AGATGACATCTCTCCGGGTCA	
	TRI12_R4	CACCCCAGGCAATTCCAAGAA	
	TRI12_F5	ATGCTCTTGTTTGGCCGTCTC	
	TRI12_R5	AGGAAAGCTGTCATCCAGGCA	
	TRI12_F6	CCTGATTCCTGGTGCAGTTTG	
	TRI12_R6	CACCCTCACGGTGCTCAATAT	
Trichothecene efflux pump (*Tri12*)	Tri12_bm_ffu_fA2	TCATCATTTCCGCAATCACTG	Gene expression
	Tri12_bm_ffu_rA2	TGGTGGTGCTTCCAAAGATGTAT	
Homologue of trichothecene 3-O-acetyltransferase (*Tri201*)	rta_tri201_F1	GCCAAAAGCAAGCTGGGCATT	Gene expression
	rta_tri201_R1	GCGACAGAATTGAGTCGAGGT	
Translation elongation factor 1-α (*TEF1A*)	rta_tef1a_F1	GGTCACTTGATCTACCAGTGC	Gene expression (reference gene)
	rta_tef1a_R1	GACATAGTAGCGAGGAGTCTC	
Ubiquitin *(UBC)*	rt_UBC_F1	TTCCCTACCGACTACCCTTTC	Gene expression (reference gene)
	rt_UBC_R1	GAGCAGATGGACAGAAGCACT	
β-tubulin *(TUB2)*	BtubF	GCCTCGACAGCAATGGTGTT	Gene expression (reference gene)
	BtubR	CCGGACTGACCGAAAACGAA	

### Annotation of Tri12 homologues

The putative *Tri12* homologues were gathered using a variant of the approach used in our previous work (Koczyk et al. 2015). Briefly, first a wide set of homologues was compiled through BLASTP searches against the combined NCBI/nr database (26/10/2015) and a local copy of Ensembl/Fungi release 28. The combined database was made non-redundant by clustering at 97% protein sequence identity with CD-HIT(Fu et al. 2012), representative sequences were inspected and kept on a per-species bases. The sequences of *Tri12* from *F. sporotrichioides* and *F. graminearum* were used as queries.

To obtain the final set of *Tri12* homologues, we performed unsupervised clustering with model transporters of known specificity. For this, the preliminary subset of candidates was combined with all available 2.A.1.3 (DHA14 antiporter family) homologues from Transporter Classification Data Base (Saier et al. 2016). The clustering was conducted in CLANS (Frickey and Lupas 2004) based on exhaustive all against all BLASTP comparisons with an expect value threshold of E-10. The stability of cluster containing *Tri12* homologues was validated at more restrictive similarity thresholds (cluster membership was tested up to 1e-80 expect value threshold). The final set, used for alignment and phylogeny reconstruction, numbered a total of 33 sequences, after also including the two sequences corresponding to protein sequence consensi of, respectively, *F. proliferatum* and *F. oxysporum Tri12* sequences obtained from the collection isolates.

We opted for the above iterative approach, as the simple selection, e.g. based on the conserved Pfam domain (TRI12) fingerprint, would result in a large set of poorly alignable distant homologues. Multiple sequences with *Tri12* similarities are, upon inspection, DHA14 transporters of completely different specificity (such as STR1—the siderophore iron transporter from *Schizosaccharomyces pombe*, Pelletier et al. 2003) or present characteristic features of multidrug transporters (e.g. SGE1*—*violet/multidrug resistance protein from *Saccharomyces cerevisiae*, Ehrenhofer-Murray et al. 1998).

Where referenced, annotation of putative transcription factor binding sites was carried out in JASPAR (Mathelier et al. 2015). Putative transmembrane elements were annotated with CCTOP (Dobson et al. 2015) and TOPCONS (Tsirigos et al. 2015).

### Sequence alignment and phylogeny reconstruction

The selected protein sequences were aligned with MAFFT-LINSI v 7.221 (Katoh and Standley 2013). For phylogenetic analysis, the multiple alignment was filtered with TCOFFEE/TCS module (Chang et al. 2014) using the transitive consistency score of 2 as the threshold (as recommended by the authors). The nucleotide sequences from the examined *F. oxysporum* (8 sequences) and *F. proliferatum* (10 sequences) isolates were aligned with MAFFT-LINSI and manually inspected for alignment correctness (referring to the earlier protein sequence alignment). For use in phylogeny reconstructions, a *F. sporotrichioides* reference *Tri12* sequence as well as additional model *F. oxysporum* (4 sequences) and *F. fujikuroi* (1 sequence) sequences were added to this alignment.

Both nucleotide and protein, maximum likelihood phylogeny reconstructions were carried out with IQTREE v 1.3.6 (Nguyen et al. 2015), using built-in model selection and ultrafast bootstrap (Minh et al. 2013) procedure. In case of nucleotide sequences, this analysis was carried out in partitioned mode, with separate models for each exon and intron (auto-selected by IQTREE).

The full alignments of both nucleotide and protein sequences, used for phylogenetic reconstructions, are included in the Supplementary Materials to this article. The alignments were visualised in CLC Genomics Workbench v 8.5.1 (Qiagen) and the phylogenetic trees were drawn with MEGA (nucleotide sequence-based tree, Tamura et al. 2013) and ETE2 (protein tree, Huerta-Cepas et al. 2010). The relevant gene structures were annotated and visualised with WebScipio (Hatje et al. 2011).

### Expression profiling

Mycelium was collected from the medium and each sample was weighed on a laboratory scale (due to rapid RNA degradation, wet weight was analysed) (Sartorius AG, Göttingen, Germany). Total RNA from chosen, representative isolates was purified using an RNeasy Plant Mini Kit (Qiagen, Hilden, Germany) according to the manufacturers’ protocol with the additional DNase digestion step. The quality of total RNA was estimated by Nanodrop (Thermo Scientific, Wilmington, DE) and via Bioanalyzer (Bio-Rad, Hercules, CA). RNA dissolved in DEPC water was stored at − 80 °C. qRT-PCR primers were designed on the basis of previously sequenced gene fragments using Primer 3 and their properties were tested using OligoCalc.

Real-time RT-PCR was used to amplify *Tri12* homologues (trichothecene efflux pump) and *Tri201* (homologue of trichothecene 3-O-acetyltransferse from *F. graminearum*) in *F. oxysporum* and *F. proliferatum* strains (Desjardins and Proctor 2007; Lee et al. 2011), and as a reference, we used housekeepeng genes *Tub2* (β-tubulin), *UBC*(ubiquitin) and *TEF*1-α (translation elongation factor) from each RNA sample of the fungal strains.

Real-time RT-PCR reactions were performed using an CFX96 Touch™ Real-Time PCR Detection System (Bio-Rad, Hercules, CA). Analyses were conducted using iTaq One Step SYBR Green RT-qPCR Kit (Bio-Rad, Hercules, California, USA). The total reaction volume was 25 μL: 12.5 μL iTaq One Step SYBR Green RT-qPCR mix, 1 μL RNA (< 35 ng), 0.5 μL each primer (10 μM), 0.125 μL reverse transcriptase and 5.125 μL nuclease free water. The reaction was carried out using the following protocol: initial denaturation 94 °C for 2 min, followed by 40 cycles at 94 °C for 15 s, 59 °C for 1 min. In the experiment, we used three biological and two technical replicates together with a template-free negative control in each analysis of both target and control genes. The melting curve analysis (from 70 to 95 °C) confirmed primer pair specificity. As a control, we used mycelium samples cultivated on medium without the addition of toxins. Relative quantification of gene expression was calculated using the 2^−ΔΔCt^ method (Bio-Rad, Hercules, CA). Data from samples treated with mycotoxin were normalised to β-tubulin, ubiquitin, 1-α translation elongation factor genes as internal controls (Real-Time PCR Application Guide, Bio-Rad, Hercules CA).

### Statistical analyses

Statistical analyses of growth patterns (relative area on the Petri dish covered by fungus) comprised analyses of variance (ANOVA) and post hoc means comparisons (Tukey-Kramer honestly significant difference [HSD]; *p* ≤ 0.05) were performed with the Statistica 9.0 software package (Stat Soft, USA). The differences in gene expression between untreated and treated samples were analysed with Wilcoxon signed-rank non-parametric test (*p* ≤ 0.05) with use of one-tailed hypothesis. The test was performed on the delta Ct values from the second day after the exposition to the indicated compound(s).

## Results

### Growth patterns of strains treated with deoxynivalenol

Among all tested isolates of *F. oxysporum* and *F. proliferatum*, the response to deoxynivalenol in concentration of 8 mg/L was weak but still significant. Greater differences were noted between species. Growth of fumonisin producing *F. proliferatum* strains was inhibited by an average of 13% and of non-producing*F. oxysporum* by 6%. Importantly, in case of *F. oxysporum* isolates, one (10 L) was significantly different (more similar to *F. proliferatum* strains) and its growth was reduced by 10% (Table 2). Addition of lower doses of mycotoxin did not result in significant growth inhibition and higher concentration of deoxynivalenol in the medium suppressed the growth of all tested strains without any exceptions.

**Table 2:** Reduction of *F. oxysporum* and *F. proliferatum* relative surface area treated with 8 mg/L of deoxynivalenol. Surface area is calculated relative to mean surface area of control samples at the fourth day of measurements

Strain	Species	Area of the colony in the comparison to the control (1%)	F statistic	*p* value	Tukey HSD Q statistic	Tukey HSD inference
10 L	*F. oxysporum*	89	2.443	0.0202	5.1073	*p < 0.05
11 L		94			0.4509	insignificant
19 L		95			0.9205	insignificant
55 L		94			2.1203	insignificant
57 L		95			0.263	insignificant
94 L		94			0.9195	insignificant
115 L		95			0.9644	insignificant
131 L		94			0.5918	insignificant
3 L	*F. proliferatum*	85	29.1237	0.0202	15.2175	*p < 0.05
7 L		84			14.5213	*p < 0.05
21 L		87			19.6747	*p < 0.05
36 L		88			11.4717	*p < 0.05
58 L		87			18.3228	*p < 0.05
59 L		86			15.3910	*p < 0.05
66 L		85			15.9318	*p < 0.05
81 L		87			15.1864	*p < 0.05
99 L		88			14.0857	*p < 0.05
1 L		89			15.2548	*p < 0.05

Growth of selected isolates in the control environment after addition of MgCl_2_, KCl, glucose, sucrose, *F. verticillioides* did not show significant changes (*p* ≤ 0.05) while the rest of the additives (ferulic acid, ground wheat seedlings, coumaric acid, H_2_0_2_, caffeine) caused significant reduction of the mycelium growth rate (Table 3).

**Table 3:** Expression (N-fold) of Tri12 homologue and reduction of mycelium area in *F. oxysporum* (mean of all tested isolates except 10 L) and *F. proliferatum* (mean of all tested isolates except 10 L) treated with different chemical and biological substances

Additive	Gene expression (N-fold)			Growth rate (mm)		
	*F. oxysporum*	*F. proliferatum*	*F. oxysporum* (10 L)	*F. oxysporum*	*F. proliferatum*	*F. oxysporum* (10 L)
Control	1.22	1.13	1.18	33.58	48.3	33.58
Deoxynivalenol	13.45*	2.92*	3.06*	31.58**	41.94**	30.18**
*F. verticillioides*	0.94	0.95	0.98	32.26	48.65	34.35
MgCI2	0.97	0.98	0.44	33.51	48.81	34.35
KCI	1.45	1.28	1.40	34.53	49.25	34.51
Ferulic acid	0.42	0.39	0.41	30.83**	42.84**	30.82**
Wheat	0.69	0.74	0.67	31.57**	45.4	29.89**
Glucose	0.73	0.69	0.75	36.6	52.65	34.02
Alert 375 SC (flusilazole)	0.46	0.52	0.47	14.10**	24.63**	15.11**
sucrose	0.89	0.85	0.91	36.6	52.65	36.13
Coumaric acid	0.29	0.35	0.31	29.81**	38.50**	29.33**
H2O2	0.31	0.29	0.33	30.74**	44.01**	30.60**
Caffeine	0.37	0.35	0.36	29.48	38.01**	29.32**

*Result is significant at *p* ≤ 0.05 (Wilcoxon signed-rank non-parametric test with use of one-tailed hypothesis)

**Result is significant at *p* ≤ 0.05 (analyses of variance (ANOVA) and post hoc means comparisons—Tukey-Kramer honestly significant difference

### Transcriptional response of Tri12 homologues in *F. oxysporum* and *F. proliferatum*

The expression of *Tri12* homologue genes was analysed in *F. oxysporum* and *F. proliferatum* strains after deoxynivalenol treatment. As a reference, *Tub2* (β-tubulin), *UBC*(ubiquitin) and *TEF*1-α (translation factor) genes were used. In the presence of mycotoxin, *Tri12* gene in *F. oxysporum* strain (11 L) has shown significantly (*P* ≤ 0.05) increased transcriptional activity (13.45-fold change–96 h after toxin exposition). Weakest induction was observed in deoxynivalenol treated *F. proliferatum* isolate, in which the relative normalised expression of *Tri12* homologue was 2.92-fold increased. Interestingly, *F. oxysporum* strain (10 L) showing similar growth patterns to the *F. proliferatum* isolates also indicated analogous *Tri12* expression (3.06-fold–96 h after toxin exposition) (Table 2). Specificity of the transcription induction of *Tri12b* homologue genes in *F. oxysporum* (trichothecene transporter) was confirmed by the profiling of *Tri12* gene expression in the presence of the multiple chemical compounds some of which can be potentially transported by the broad specific efflux pumps (Table 3). Only addition of deoxynivalenol significantly increased expression of *Tri12* gene (13.45-fold). In the case of the rest used substances, addition of potentially harmful ferulic acid, coumaric acid, fungicide (Alert 375 SC), caffeine, H_2_O_2_ caused significant (*P* < 0.05) decrease of transcript level. Addition of sugars (glucose, sucrose), microelements (MgCl_2_, KCl) and potential host tissues (wheat leaves and roots) did not influence gene expression.

### Transcriptional response of Tri201 homologues in *F. oxysporum* and *F. proliferatum*

To analyse general response of *F. oxysporum* and *F. proliferatum* strains to the presence of deoxynivalenol in the environment, expression of *Tri201* gene was tested (*Tri201* is a homologue of *Tri101*gene—responsible for detoxification by 3-O-acetylation of the trichothecene skeleton in the biosynthetic pathway in *F. graminearum*). In the presence of mycotoxin, *Tri201* gene in *F. oxysporum* strains has shown significantly (*P* ≤ 0.05) increased transcriptional activity (13.07-fold–96 h after toxin exposition). No induction was observed in deoxynivalenol-treated*F. proliferatum* isolates.

### Sequence and phylogeny of Tri12 homologues

**Fig. 1:**
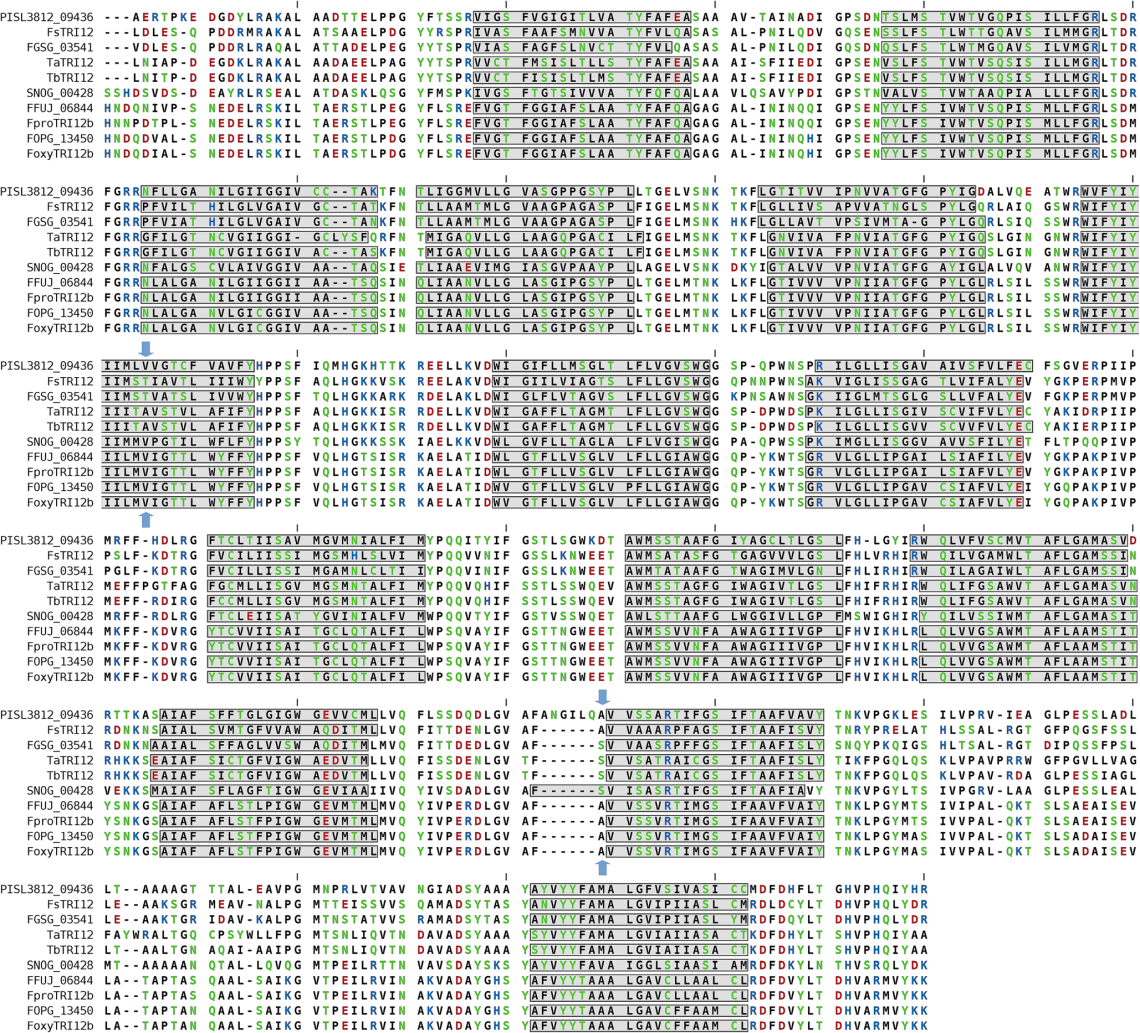
Alignment of protein sequences arising from the translation of divergent *Tri12* homologues shows conserved structural features. Grey rectangles correspond to consensus transmembrane region predictions, as predicted by TOPCONS and CCTOP. Blue arrows mark the conserved positions of splicing sites (based on gene models from Ensembl/Genbank corresponding nucleotide sequences). The poorly alignable N- and C-terminal regions were truncated. Sequences shown: PISL3812_09436—*Talaromyces islandicus Tri12* homologue; FGSG_03541—*F. graminearum Tri12*; FsTRI12—*F. sporotrichioides Tri12* (NCBI: AAK33071); TaTRI12—*T. arundinaceum Tri12* (NCBI: CAY87358); TbTRI12—*T. brevicompactum Tri12* (NCBI: CCA31154); SNOG_00428—*Stagonospora nodorum Tri12* homologue; FFUJ_06844—*F. fujikuroi Tri12* homologue; FproTRI12b—consensus protein sequence of *Tri12* homologues from the analysed *F. proliferatum* isolates; FoxyTRI12b—consensus protein sequence of the *Tri12* homologues from the analysed *F. oxysporum* isolates; FOPG_13450—*Fusarium oxysporum* f. sp. conglutinans *Tri12* homologue

**Fig. 2:**
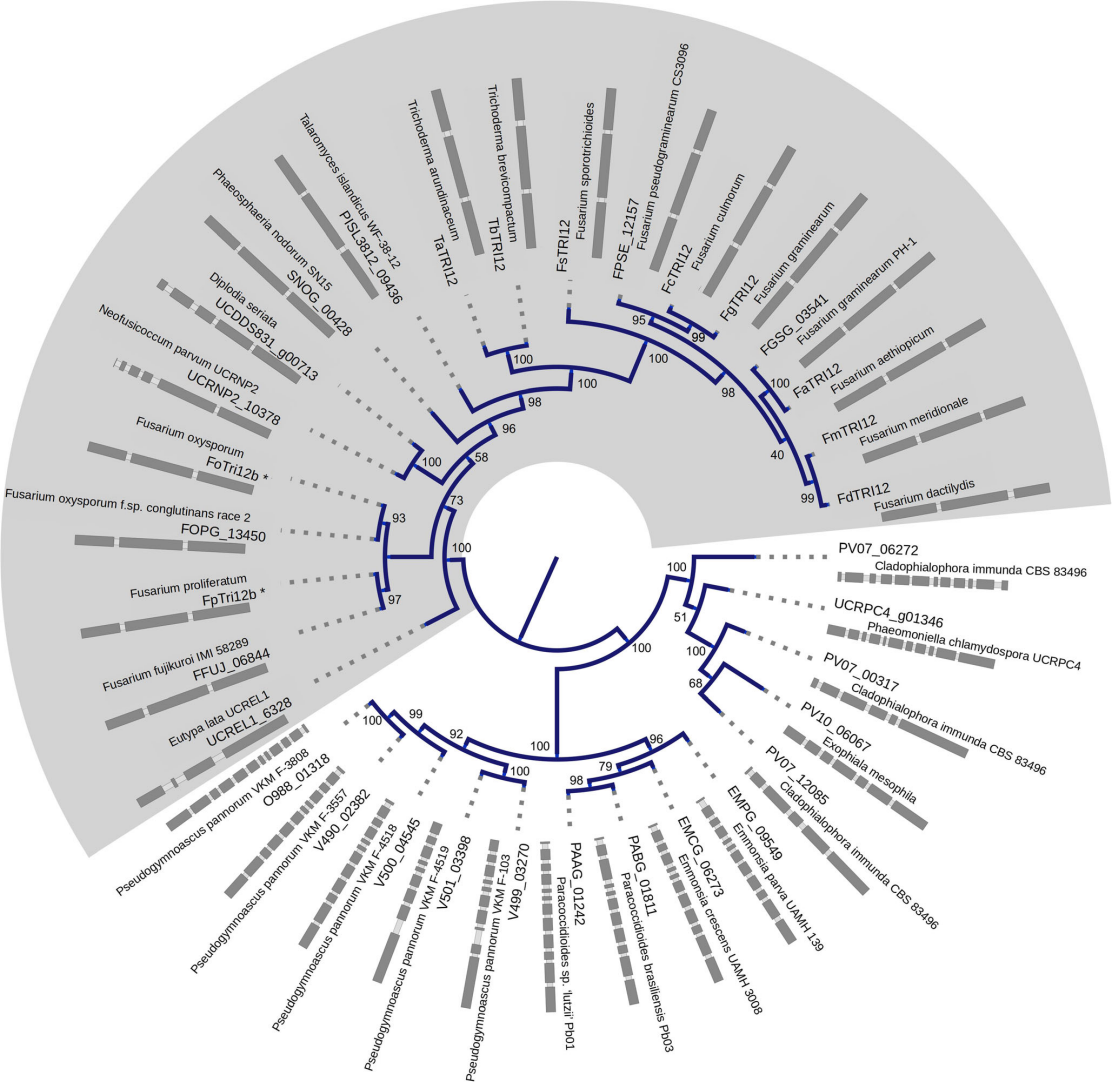
Extended majority rule consensus tree (maximum likelihood reconstruction) for non-redundant set of *Tri12* homologues from different species (clustered at 97% protein sequence identity with CD-HIT, with exception of Fp and FoTRI12b sequences which represent consensus sequences for examined isolates). Scale is in amino acid residue change per site. Intron-exon structure is visualised (WebScipio remapping of original protein and nucleotide sequences). The tree was created with IQTREE v 1.3.6 (multi-threaded) using ultrafast bootstrap with automated stopping criterion based on topology convergence. The analysis was run with LG + G + F model (auto-selected). Following abbreviations were used for sequences obtained from NCBI: TaTRI12 (*Trichoderma arundinaceum*, CAY87358), TbTRI12 (*T.* brevicompactum, CCA31154), FsTRI12 (*Fusarium sporotrichioides*, AAK33071), FaeTRI12 (*F. aethiopicum*, ACJ69853), FcTRI12 (*F. culmorum*, AAM48786), FgTRI12 (*F. graminearum*, BAA76934), FmTRI12 (*F. meridionale*, AAM48906), FdTRI12 (*F. dactylidis*, AJC98152), other sequences were obtained from Ensembl/Fungi (v28) and are listed under their respective locus locus designations

The protein sequences of *Tri12* cluster members were clearly alignable with conserved transmembrane regions and (to a degree—Figs. 1 and 2) conserved splice junction positions in relation to the multiple sequence alignments.

The subsequent phylogenetic reconstruction of evolutionary relationships between *Tri12* homologues (Fig. 2) has confirmed the distant relationship between canonical *Tri12* genes present in sambucinum complex fusaria and more distant homologues (referred to as *Tri12b*) found in *oxysporum* and *fujikuroi* complexes. Majority of bipartitions were strongly supported (> 70% support) in ultrafast bootstrap analysis.

However, the attempts to root the resulting trees with even more distant homologues from DHA14 subset of MFS1 transporters have led to inconsistent results. This is likely due to ‘twilight zone’ (Rost 1999) levels of sequence similarity (around 20% protein sequence identity; BLAST expect values < 1e-20) in comparison to the considered outgroups (the sequences from 2.A.1.3 level of TCDB classification of transporters, e.g. *Mfs1* from *T. harzianum*, *Vba5p* from *Saccharomyces cerevisiae*). Thus, we have opted for midpoint rooting in our reconstruction of the *Tri12* ancestry (Hess and De Moraes Russo 2007).

**Fig. 3:**
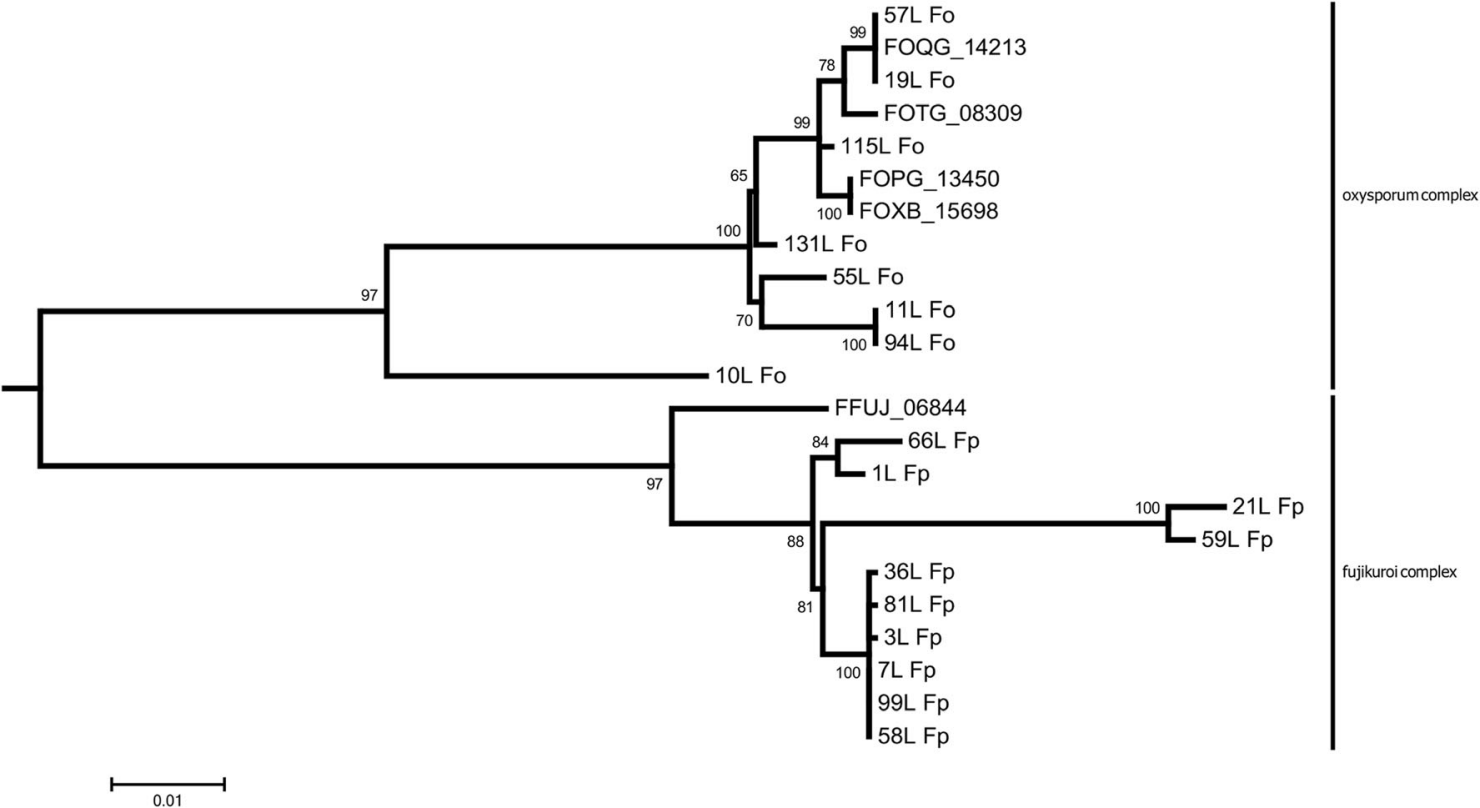
Majority rule consensus tree for *Tri12b* nucleotide sequences of examined *F. oxysporum* and *F. proliferatum* isolates (maximum likelihood reconstruction; bipartititions with support less than 50% were collapsed). Scale is in nucleotide changes per site. Both exon and intron sequences were used in the reconstruction—total of 1849 aligned positions. *F. sporotrichioides Tri12* was used as outgroup to root the tree (not shown). The tree was created with IQTREE v 1.3.6 (multi-threaded) using ultrafast bootstrap with automated stopping criterion based on topology convergence. The analysis was run in partitioned mode, with separate models predicted for each exon and intron (respectively, exon 1–K2P + G, exon 2–TPM3 + G, exon 3–K2P + I, intron 1–K3P, intron 2–K2P). Additional *Tri12* homologues from model genomes shown: FOQG_14213–*F. oxysporum* f. sp. raphani, FOTG_08309–*F. oxysporum* f. sp. vasinfectum, FOPG_13450–*F. oxysporum* f. sp. conglutinans, FOXB_15698*–F. oxysporum* Fo5176 (isolated from *Arabidopsis thaliana*), FFUJ_06844–*F. fujikuroi*

**Fig. 4:**
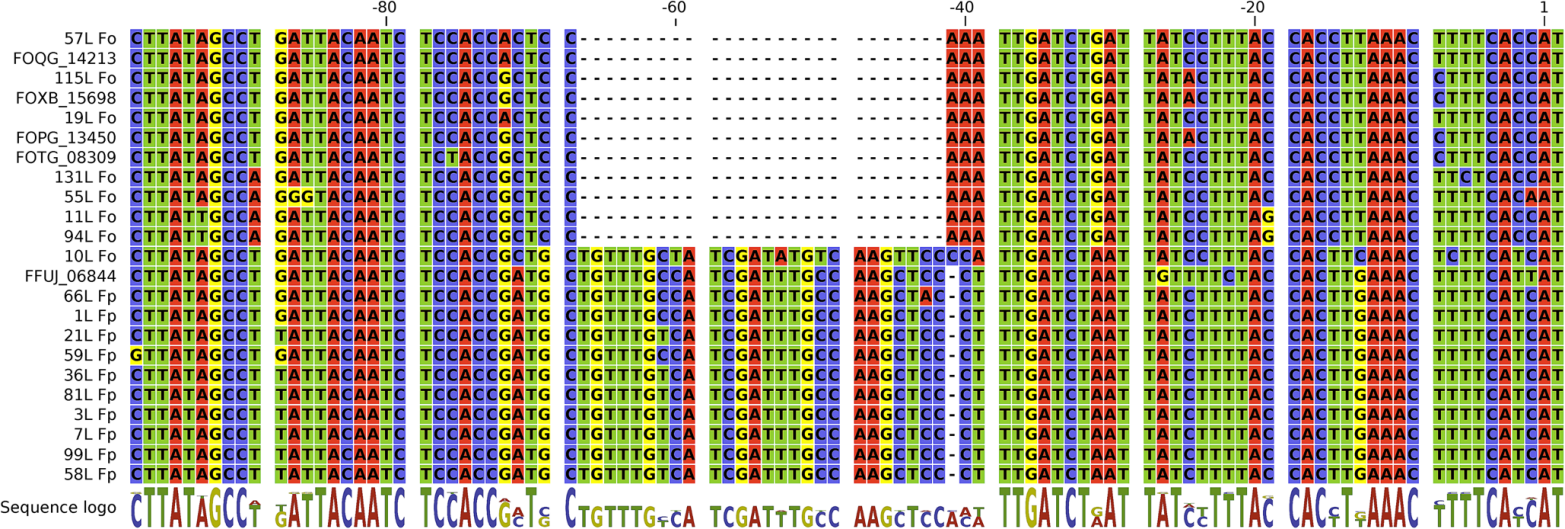
Alignment of pregenic sequence (ca. 100 base pairs) of examined *F. oxysporum* (Fo) and *F. proliferatum* (Fp) isolates shows a conserved motif (associated with lower gene expression) present in *F. proliferatum* and a single, early diverging *F. oxysporum* isolate (10 L). The ordering and included sequences from model genomes are same as on the Fig. 3

Nucleotide sequence comparisons between the *F. oxysporum* and *F. proliferatum* isolates have shown their monophyleticity as members of their respective species complexes (Fig. 3—maximum likelihood tree). The sequence alignments have also uncovered a 25-26 bp indel differentiating between *F. oxysporum* and *F. fujikuroi*/*proliferatum* promotor regions (Fig. 4). We posit that this difference is possibly tied to the observed divergence in expressional patterns (see the following section for details). Interestingly, in *F. oxysporum* strain (10 L) showing similar growth patterns to the *F. proliferatum* isolates, sequence alignments have also uncovered a similar indel (Fig. 4). All *Tri12* homologue sequences are available in NCBI database (KX273324, KX273325, KX273326, KX273327, KX273328, KX273329, KX273330, KX273331, KX273332, KX273333, KX273334, KX273335, KX273336, KX273337, KX273338, KX273339, KX273340, KX273341).

## Discussion

Since the initial discovery and experimental characterisation of Tri12 efflux pomp (Alexander et al. 1999), subsequent inquiries have established its role in self-protection and virulence of trichothecene-producing strains (Menke et al. 2012). However, while the transformative detoxification mechanism in the form of *Tri101*O-3-acetyltransferase was found to be crucial in trichothecene resistance in multiple producing and non-producing species, the deletion experiments pointed to less significant role of the active efflux (Kimura et al. 2007; Khatibi et al. 2011). The presence and possible involvement of *Tri12* homologues in non-producing species were largely left uninvestigated.

Our phylogeny reconstruction results (see Fig. 2) support early divergence of canonical *Tri12* homologues in the *Fusarium* genus (present mostly in the *sambucinum* complex of the genus, as well as *Trichoderma arundinaceum* and *T. brevicompactum*) and the putative trichothecene transporter *Tri12b* (present in the *oxysporum* and *fujikuroi* complexes, with a notable exception of *F. verticillioides—*see Figs. 2 and 3). The phylogenetic placement of *TaTri12* and *TbTri12* points to a split predating the divergence of both genii and presence of additional homologues in *Dothideomycetes* (*Diplodia seriata*, *Neofusicoccum parvum*, *Stagonospora nodorum*) and *Eurotiomycetes* (*Talaromyces islandicus*) possibly substantiates either an even more distant relationship or possible spread via horizontal gene transfer.

During the preparation of the final stages of this work, a comprehensive analysis of the evolutionary origins of structural diversity in trichothecenes was put forth by Proctor and colleagues (Proctor et al. 2018). Based on multiple venues of evidence (trichothecene biosynthesis/metabolism-related phylogenies, chemical analyses and functional genomics evidence), the authors have strongly corroborated the diverse and discontinuous distribution of trichothecene biosynthetic capability among ascomycetes (in particular divergent *Hypocreales* species, as well as the *incertae sedis* ascomycete-*Microcyclospora tardicrescens*). The different scenarios implied by branching of gene histories in *Nectriaceae*, *Cordycipitaceae* and *Hypocreaceae* families underscore the possibility of both ancestral duplications and horizontal transfer between diverged donors/acceptors. Notably, in regards to TRI12, the conclusions have highlighted both optional role of the transporter in self-resistance of producers and possibility of compensatory role of different transporters with overlapping affinities for toxic compounds.

While the effects of trichothecenes on plant (Ohsato et al. 2007; Walter et al. 2010) and bacterial (Bisht et al. 2013) growth were previously characterised, the exact effect of the toxin on the growth of non-producing fusaria was, to our knowledge, previously not quantified. Through growth assays, we found that on the average the effects of trichothecene toxins on other fusaria are slight but statistically significant. On the average, *Fusarium oxysporum* isolates were found to be more resistant than *F. proliferatum*, with an exception of *F. oxysporum* 10 L. We found that the variation in resistance could likely be attributed to differences in the *TRI12b* homologue promoter region and overall transcriptional response (see also the following sections).

Our analysis of expression patterns (see Table 3) shows that *F. oxysporum* undergoes rapid shift in *Tri12b* expression upon trichothecene treatment. This reaction is not as strong in *F. proliferatum* and a divergent *F. oxysporum* isolate 10 L. Since no such response was observed on treatment with multiple different stressors (those agents that caused decreased growth also reduced expression of *Tri12* probably due to the overall effect on fungal metabolism), we conclude that it should be considered as indirect evidence for *Tri12b* involvement, as an efflux pump contributing to the observed *F. oxysporum* resistance to trichothecene toxins. Additionally in the presence of mycotoxin, *Tri201* gene (*Tri201* is a homologue of *Tri101*gene—responsible for detoxification by 3-O-acetylation of the trichothecene skeleton in the biosynthetic pathway in *F. graminearum*) in *F. oxysporum* strains has also shown increased transcriptional activity whereas no induction was observed in deoxynivalenol treated *F. proliferatum* isolates.

The analysis of the promoter region of *F. proliferatum* and *F. oxysporum* isolates shows that this difference could possibly arise from a 25–26 bp deletion observed across all other *oxysporum* isolates. The cross-referencing with JASPAR-FUNGI database of known regulatory motifs shows that this stretch encodes possible pH-related TF binding site capable of binding PACC/RIM101 transcription factor (Supplementary Table 1). Deletion in this case would serve to decouple transporter expression from indirect environmental cues in form of pH (beneficial for a resistant non-producer) which has been previously documented to influence the expression of genes found in the canonical trichothecene cluster (Merhej et al. 2011) and virulence (Caracuel et al. 2003).

Notably, both predicted protein (aligned TM regions—Fig. 1) and gene structures (two introns of conserved position in regards to protein sequence—Figs. 1 and 2) are heavily conserved between both fusarial clades. Genes with rapid fluctuations are known to possess fewer exons, due to energetic costs associated with multiplicity of splicing events (Jeffares et al. 2008). As a toxin-associated efflux pump involved in early response *Tri12b* would thus be well tailored for rapid transcriptional response observed in the isolates of *F. oxysporum*.

## Electronic supplementary material

ESM 1 (DOC 236 kb)
