# Supplementary material for: Efflux pumps as an additional source of resistance to trichothecenes in Fusarium proliferatum and Fusarium oxysporum isolates
Source: J Appl Genet. 2019 Jun 27;60(3):405–16. doi: 10.1007/s13353-019-00501-2 (PMC6803570; doi:10.1007/s13353-019-00501-2)
Supplement: Supplementary file 1 — (DOC 236 kb) [file 13353_2019_501_MOESM1_ESM.doc]

**Supplementary Table 1.** Putative transcriptional factor binding sites annotated based on JASPAR CORE FUNGI subset at 80% sequence identity threshold.

| **Model name** | **Factor description according to JASPAR** | **Relative score** | **Start** | **End** | **Strand** | **predicted site sequence** |
| --- | --- | --- | --- | --- | --- | --- |
| *FKH2* | Forkhead family transcription factor; plays a major role in the expression of G2/M phase genes; positively regulates transcriptional elongation | 0,86 | 1 | 7 | -1 | GCAAACA |
| *RFX1* | Major transcriptional repressor of DNA-damage-regulated genes | 0,85 | 2 | 9 | 1 | GTTTGCCA |
| *TOS8* | Homeodomain-containing protein and putative transcription factor; induced during meiosis and under cell-damaging conditions | 0,82 | 3 | 10 | -1 | ATGGCAAA |
| *YAP5* | Basic leucine zipper (bZIP) iron-sensing transcription factor; involved in diauxic shift | 0,84 | 4 | 9 | -1 | TGGCAA |
| *MOT3* | Transcriptional repressor (in hypoxia) and activator (in aerobic conditions) with two C2-H2 zinc fingers | 0,82 | 5 | 10 | -1 | ATGGCA |
| *XBP1* | Transcriptional repressor; induced by stress and starvation during mitosis | 0,82 | 8 | 14 | -1 | ATCGATG |
| *SPT23* | ER membrane protein involved in regulation of *OLE1* transcription | 0,81 | 10 | 17 | -1 | CAAATCGA |
| *YPR013C* | Uncharacterised; putative zinc finger protein | 0,88 | 11 | 19 | -1 | GGCAAATCG |
| *GLN3* | Transcriptional activator in nitrogen starvation. Regulates genes regulated by nitrogen catabolite repression. | 0,86 | 12 | 16 | 1 | GATTT |
| *RFX1* | Major transcriptional repressor of DNA-damage-regulated genes | 0,80 | 13 | 20 | 1 | ATTTGCCA |
| *YAP5* | Basic leucine zipper (bZIP) iron-sensing transcription factor; involved in diauxic shift | 0,84 | 15 | 20 | -1 | TGGCAA |
| *MOT3* | Transcriptional repressor (in hypoxia) and activator (in aerobic conditions) with two C2-H2 zinc fingers | 0,82 | 16 | 21 | -1 | TTGGCA |
| *RIM101* | Cys_2_His_2_ zinc-finger transcriptional repressor; involved in alkaline responsive gene repression | 1,00 | 16 | 22 | 1 | TGCCAAG |
| *HAP2* | Subunit of the Hap2p/3p/4p/5p CCAAT-binding complex; complex is heme-activated and glucose-repressed and acts as a transcriptional activator | 0,99 | 17 | 21 | -1 | TTGGC |

**Supplementary Data 2.** Full alignment of protein sequences (FASTA format) corresponding to the phylogeny of *Tri12* homologs from different fungal species (Figure 2, main manuscript, only columns with TCS score>2 were taken into account during reconstruction).

| >PISL3812_09436  M-------------------ASTHDPS------------AERTPKEDGDYLRAKALAADT  T----ELPPGYFTSSRVIGSFVGIGITLVATYFAFEASAAAV-TAINADI----------  -------GPSD--NTSLM------STVWTVG-----QPISILLFGRLSDRFGRRNFLLGA  NILGIIGGIVCC--TAKTFNTLIGGMVLLGVASGPPGSYPLLTGELVSNKTKFLGTITVV  IPNVVATGFGPYIGDALVQE-ATWRWVFYIYIIMLVVGTCFVAVFYHPPSFIQMHGKHTT  KREELLKVDWIGIFLLMSGLTLFLVGVSWGGSP--QPWNSPRILGLLISGAVAIVSFVLF  ECFSGVERPIIPMRFF-HDLRGFTCLTIISAVMGVMNIALFIMYPQQITYIF--GSTLSG  WKDTAWMSSTAA----FGIYAGCLTLGSLFH-LGYIRWQLVFVSCMVTAFLGAMASVDRT  TKASAIAFSFFTGLGIGWGEVVCMLLVQFLSSDQDLGVAFANGILQAVVSSARTIFGSIF  TAAFVAVYT-----------------NKVPGKLESILVPRV-IEAGLPESSLADLLT--A  AAAGTTTAL-EAVPGM--NPRLVTVAVNGIADSYAAAYAYVYYFAMALGFVSIVASICCM  DFDHFLTGHVPHQIYHRKETEVDPLGSRTELDVNMKSGE------DQKV-----------  ---------------  >TaTRI12  MG--------------------APDEV------------LNIAP-DEGDKLRAKALAADA  E----ELPAGYYTSPRVVCTFMSISLTLLSTYFAFEASAAAI-SFIIEDI----------  -------GPSE--NVSLF------STVWTVS-----QSISILLMGRLTDRFGRRGFILGT  NCVGIIGGI-GCLYSFQRFNTMIGAQVLLGLAAGQPGACILFIGELMSNKTKFLGNVIVA  FPNVIATGFGPYIGQSLGIN-GNWRWIFYIYIIITAVSTVLAFIFYHPPSFAQLHGKKIS  RRDELLKVDWIGAFFLTAGMTLFLLGVSWGGSP--DPWDSPKILGLLISGIVSCVIFVLY  ECYAKIDRPIIPMEFFPGTFAGFGCMLLISGVMGSMNTALFIMYPQQVQHIF--SSTLSS  WQEVAWMSSTAG----FGIWAGIVTLGSLFHIFRHIRWQLIFGSAWVTAFLGAMASVNRH  KKSEAIAFSICTGFVIGWAEDVTMLLVQFISSDENLGVTF------SVVSATRAICGSIF  TAAFISLYT-----------------IKFPGQLQSKLVPAVPRRWGFPGVLLVAGFAYWR  ALTGQCPSYWLLFPGM--TSNLIQVTNDAVADSYAAAYSYVYYFAMALGVIAIIASACTK  DFDHYLTSHVPHQIYAAKDADVDLLDS----DRSTENVSSAAVTVSEKE-----------  ---------------  >TbTRI12  MG--------------------APDEV------------LNITP-DEGDKLRAKALAADA  E----ELPAGYYTSPRVVCTFISISLTLMSTYFAFEASAAAI-SFIIEDI----------  -------GPSE--NVSLF------STVWTVS-----QSISILLMGRLTDRFGRRGFILGT  NCVGIIGGIVAC--TASKFNTMIGAQVLLGLAAGQPGACILFIGELMSNKTKFLGNVIVA  FPNVIATGFGPYIGQSLGIN-GNWRWIFYIYIIITAVSTVLAFIFYHPPSFAQLHGKKIS  RRDELLKVDWIGAFFLTAGMTLFLLGVSWGGSP--DPWDSPKILGLLISGVVSCVVFVLY  ECYAKIERPIIPMEFF-RDIRGFCCMLLISGVMGSMNTALFIMYPQQVQHIF--SSTLSS  WQEVAWMSSTAG----FGIWAGIVTLGSLFHIFRHIRWQLIFGSAWVTAFLGAMASVNRH  KKSEAIAFSICTGFVIGWAEDVTMLLVQFISSDENLGVTF------SVVSATRAICGSIF  TAAFISLYT-----------------NKFPGQLQSKLVPAV-RDAGLPESSIAGLLT--A  ALTGNAQAI-AAIPGM--TSNLIQVTNDAVADSYAAAYSYVYYFAMALGVIAIIASACTK  DFDHYLTSHVPHQIYAAKDADVDLLDS----DRSTKNVSSSAVTVSEKE-----------  ---------------  >FsTRI12  M------------------TVVVPEEG------------LDLES-QPDDRMRAKALATSA  A----ELPDGYYRSPRIVASFAAFSMNVVATYFVLQASASAL-PNILQDV----------  -------GQSE--NSSLF------STLWTTG-----QAVSILMMGRLTDRFGRRPFVILT  HILGLVGAIVGC--TATKFNTLLAAMTMLGVAAGPAGASPLFIGELMSNKTKFLGLLIVS  APVVATNGLSPYLGQRLAIQ-GSWRWIFYIYIIMSTIAVTLIIIWYYPPSFAQLHGKKVS  KREELAKVDWIGIILVIAGTSLFLLGVSWGGQPN-NPWNSAKVIGLISSGAGTLVIFALY  EVYGKPERPMVPPSLF-KDTRGFVCILIISSIMGSMHLSLVIMYPQQVVNIF--GSSLKN  WEETAWMSATAS----FGTGAGVVVLGSLFHLVRHIRWQILVGAMWLTAFLGAMSSINRD  NKNSAIALSVMTGFVVAWAQDITMLLVQFITTDENLGVAF------AVVAAARPFAGSIF  TAAFISVYT-----------------NRYPRELATHLSSAL-RGTGFPQGSFSSLLE--A  AKSGRMEAV-NALPGM--TTEISSVVSQAMADSYTASYANVYYFAMALGVIPIIASLCMR  DLDCYLTDHVPHQLYDRKNAHKDVLEG----NSESQPSPIILSMADKE------------  ---------------  >FGSG_03541  M------------------TATVHEKG------------VDLES-QPDDRLRAQALATTA  D----ELPEGYYTSPRVIASFAGFSLNVCTTYFVLQASASAL-PNILQDI----------  -------GQSD--NQSLF------STLWTMG-----QAVSILVMGRLTDRFGRRPFVIAT  HILGLVGAIVGC--TANKFNTLLAAMTMLGVAAGPAGASPLFIGELMSNKHKFLGLLAVT  VPSIVMTA-GPYLGQRLSIQ-SSWRWIFYIYIIMSTVATSLIVVWYHPPSFTQLHGKKAR  KRDELAKLDWIGLFLVTAGVSLFLLGVSWGGKPN-SAWNSGKIIGLMTSGLGSLLVFALY  EVFGKPERPMVPPGLF-KDTRGFVCILLISSIMGAMNLCLTIIYPQQVINIF--GSSLKN  WEETAWMTATAA----FGTWAGIMVLGNLFHLIRHIRWQILAGAIWLTAFLGAMSSINRD  NKNAAIALSFFAGLVVSWAQDITMLMVQFITTDEDLGVAF------SVVAASRPFFGSIF  TAAFISLYS-----------------NQYPKQIGSHLTSAL-RGTDIPQSSFPSLLE--A  AKTGRIDAV-KALPGM--TNSTATVVSRAMADSYTASYANVYYFAMALGVIPIIASLYMR  DFDQYLTDHVPHQLYDRNKADKDVLEG----DSDSQSSPTILSIVDDKTQRYFHSSIGVT  SRKTALTDDTAKI-S  >FPSE_12157  M------------------TATVPKEG------------VDLES-QPGDRLRAQALATTA  A----ELPEGYYTSPRVIASFAAFSLNVCATYFVLQASASAL-PNILQDI----------  -------GQSD--NQSLF------STLWTTG-----QAVSILVMGRVTDRFGRRPFVIAT  HILGLVGAIVGC--TANKFNTLLAAMTLLGVAAGPAGSSPLFIGELMSNKHKFLGLLAVS  APSIVMTA-GPYFGQRLSIQ-SSWRWIFYIYIIMSAVATLLIVVWYHPPSFRQLHGKKAR  KRDELAKLDWIGIFLVTAGVSLFLLGVSWGGKPN-NPWNSGKIIGLMTSGLGSLVVFALY  EVFGKPVQPMIPPVLF-KDTRGFVCILLISSIMGAMNLCLTIIYPQQVINIF--GSSLKN  WQETAWMTATAS----FGTWAGVIILGNVFHLIRHIRWQILVGAMWLTAFLGAMSSVNRD  NKNAAIALSFFSGFVVGWAQDITMLMVQFITTDEDLGVAF------SVVAASRPFFGSIF  TAVFISLYS-----------------NQYPKEIGSHLTSAM-RGTDIPQASFPSLLE--A  AKTGRIDAV-KALPGM--TNSTTTVVSQAMADSYTASYANVYYFAMALGVIPIIASLCMR  DFDRYLTDHVPHQLYDRKKADKDVLEG----DSDTPSSPTIHSTVEVKE-----------  ---------------  >FaeTRI12  M------------------AATVPKEG------------VDLES-QPDDRLRAQALATTA  A----ELPEGYYRSPRVIASFAAFSLNVCATYFVLQASASAL-PNILQDI----------  -------GQSN--NQSLF------STLWTMG-----QAVSILVMGRLTDRFGRRPFVIAT  HILGLVGAIVGC--TANKFNTLLAAMTMLGFAAGPAGASPLFIGELMSNKHKFLGLLAVS  VPSIVMTA-GPYLGQRLSIQ-SSWRWIFYIYIIMSTVATSLIVVWYHPPSFTQLHGKKAR  KRDELAKLDWIGLFLVTAGVSLFLLGVSWGGKPN-SAWDSGKIIGLMTSGLGSLLVFALY  EVFGKPERPMVPPGLF-KDTRGFVCILLISSIMGAMNLCLTIIYPQQVINIF--GSSLKN  WEETAWMTATAA----FGTWAGIMVLGNLFHLVRHIRWQILAGAMWLTAFLGAMSSINRN  NKNAAIALSFFAGLVVSWAQDITMLMVQFITTDEDLGVAF------SVVAASRPFFGSIF  TAAFISLYS-----------------NQYPKEIGGHLTSAL-RGTDIPQSSFPSLLE--A  AKTGRIDAV-KALPGM--TNSTATVVSRAMADSYTASYANVYYFAMALGVIPIIASLYMR  DFDQYLTDHVPHQLYDRNKADKDVLGG----DSDSQSSPTIHSIVEDKK-----------  ---------------  >FcTRI12  M------------------TASVHEKG------------VDLES-QPDDRLRAQALATTA  D----ELPEGYYTSPRVIASFAGFSLNVCSTYFVLQASASAL-PNILQDI----------  -------GQSE--NQGLF------STLWTMG-----QAVSILVMGRLTDRFGRRPFVIAT  HIIGLVGAIVGC--TTNKFNTLLAAMTLLGVAAGPAGSSPLFVGELMSNKTKFLGLLTVS  IPTIVMSA-GPYFGQRLSIQ-GNWRWIFYIYIIMSAIAVLLIVVWYHPPSFRQLHGKKAR  KRDELAKLDWIGIFLVSVGVSLFLLGVSWGGKPN-SPWNSSKIIGLMTSGLGSLVVFALY  EVFGKPVQPMIPPALF-KDTRGFVCILLISSIMGAMNLCLTIIYPQQVINIF--GSSLKN  WQETAWMTATAS----FGTWAGVMILGNVFHLIRHIRWQILVGALWLTAFLGAMSSVNRD  NKNAAIALSFFSGFVVGWAQDITMLMVQFITTDEDLGVAF------SVVAASRPFFGSIF  TAAFISLYS-----------------NQYPKEIGSHLTSAM-RGTDIPQASFPSLLE--A  ATTGRIDAV-KALPGM--TNSIAAVVSRAMADSYTASYANVYYFAMALGVIPIIASLCMR  DFDRYLTDHVPHQIYDRKKADKDVLDG----DSDTPSSPIIHSTVEVKE-----------  ---------------  >FgTRI12  M------------------TATVHEKG------------VDLES-QPDDRLRAQALATTA  D----ELPEGYYTSPRVIASFAGFSINVCATYFVLQASASAL-PNILQDI----------  -------GQSE--NQGLF------STLWTMG-----QAVSILVMGRLTDRFGRRPFVIAT  HIIGLVGAIVGC--TAKEFNTLLAAMTLLGVAAGPAGSSPLFVGELMSNKTKFLGLLTVT  IPTIIMSA-GPYFGQSLSIQ-GNWRWIFYIYIISSAVAALLIVVWYHPPSFRQLHGKKAR  KRDELAKLDWIGIFLVTAGVSLFLLGVSWGGKPN-NPWNSGKIIGLMTSGLGSLVVFALY  EVFGKPVQPIIPPVLF-KDTRGFVCILLISSIMGAMNLCLTILYPQQVINIF--GSSLKN  WQETAWMTATAS----FGTWAGVMILGNVFHLIRHIRWQILVGAMWLTAFLGAMSSVNRD  NKNAAIALSFFSGFVVGWAQDITMLMVQFITTDEDLGVAF------SVVAASRPFFGSIF  TAVFISLYS-----------------NQYPKEIGSHLTSAM-RGTDIPQASFPSLLE--A  AKTGRIDAI-KALPGM--TNSTATVVSQAMADSYTASYANVYYFAMALGVIPIIASLCMR  DFDRYLTDHVPHQIYDRKKADKDVLEG----DSDTPSSSTIHSTIEVKA-----------  ---------------  >FmTRI12  M------------------TATVPQEGV-----------VDLES-QPEDRLRAEALATTA  A----ELPEGYYTSARVMASFAAFSLNVCATYFVLQASASAL-PNILQDI----------  -------GQSE--NESLF------STLWTTG-----QAVSILVMGRLTDRFGRRPFVIAT  HILGLVGAIVGC--TANKFNTLLAAMTMLGVAAGPAGASPLFIGELMSNKTKFLGLLVVS  GPNIVANM-GPYFGQRLSIE-GDWRWIFYIYIIISAIATVLIVVWYHPPSFTQLHGKKAR  KRDELAKLDWIGIFLVTAGVSLFLLGVSWGGKPN-SAWDSGKIIGLMTSGLGSLLVFALY  EVYGKPERPMVPPALF-KDFRGFVCILLISSIMGAMNLCLTIIYPQQVINIF--GSSLKN  WQETAWMTATAS----FGTWTGVMILGNVFHLIRHIRWQILVGAMWLTAFLGAMSSVNRH  NKNAAIALSFFSGFVVAWAQDITMLMVQFITTDEDLGVAF------SVVAASRPFFGSIF  TAVFISLYS-----------------NQYPKEIGSHLTSAL-RGTDIPQSSFPSLLE--A  AKTGRIDAV-KALPGM--TNSTAAVVSGAMADSYTASYANVYYFAMALGVIPIIASLCMK  NFDQYLTDHVPHQLYDRKKADKDVLEG----DSDSQSSPTIHSIVEDKK-----------  ---------------  >FdTRI12  M------------------TVTVPQEG------------VDLES-QPDDRLRAQALATTA  A----ELPEGYYTSPRVIASFAAFSLNVCATYFVLQASASAL-PNILQDI----------  -------GQSE--NESLF------STLWTTG-----QAVSILVMGRLTDRFGRRPFVIAT  HILGLVGAIVGC--TANEFNTLLAAMTMLGVAAGPAGASPLFIGELMSNKTKFLGLLVVS  GPNIVANM-GPYFGQRLSIQ-GNWRWIFYIYIIISAIATVLIVVWYHPPSFTQLHGKKAR  KRDELAKLDWIGIFLVTAGVSLFLLGVSWGGKPN-SAWDSGKIIGLMTAGLGSLVVFALY  EVYGKPERPMVPPALF-KDTRGFVCILLISSIMGAMNLCLTIIYPQQVINIF--GSSLKN  WQETAWMTATAS----FGTWTGVMILGNVFHLIRHIRWQILVGAMWLTAFLGAMSSVNRN  NKNAAIALSFFSGFVVAWAQDITMLMVQFITTDEDLGVAF------CMYS---------Y  LYCWDISNN-----------------NQYPKEIGSHLTSAL-RGTDIPQSSFPSLLE--A  AKTGRIDVV-KALPGM--TKSTAAVVSRAMADSYTASYANVYYFAMALGVIPILASLCMK  NFDQYLTDHVPHQLYDRKKADKDILEG----DSDSQSSPTIHSIVEDKK-----------  ---------------  >SNOG_00428  MAD----------------MAPPTKESSEKPPREHASSHDSVDS-DEAYRLRSEALATDA  S----KLQSGYFMSPKIVGSFTGTSIVVVATYFQFQALAAVLQSAVYPDI----------  -------GPST--NVALV------STVWTAA-----QPIALLLFGRLSDRFGRRNFALGS  CVLAIVGGIVAA--TAQSIETLIAAEVIMGIASGVPAAYPLLAGELVSNKDKYIGTALVV  VPNVIATGFGAYIGLALVQV-ANWRWIFYIYIIMMVPGTILWFLFYHPPSYTQLHGKKSS  KIAELKKVDWLGVFLLTAGLALFLVGISWGGPA--QPWSSPKIMGLLISGGVAVVSFILY  ETFLTPQQPIVPMRFF-RDLRGFTCLEIISATYGVINIALFVMWPQQVTYIF--GSTVSS  WQETAWLSTTAA----FGLWGGIVLLGPFMSWIGHIRYQILVSSIWMTAFLGAMASITVE  KKSMAIAFSFLAGFTIGWGEVIAAIIVQYIVSDADLGVAF------SVISASRTIFGSIF  TAAFIAVYT-----------------NKVPGYLTSIVPGRV-LAAGLPESSLEALMT--A  AAAANQTAL-LQVQGM--TPEILRTTNVAVSDAYSKSYAYVYYFAVAIGGLSIAASIAMR  DFDKYLNTHVSRQLYDKKDAHTDPLED----AGMSHGVGTRDND-GEKAV----------  --------------V  >FFUJ_06844  ----------------------MTKQEASPDDISPGHNDQNIVP-SNEDELRSKILTAER  S----TLPEGYFLSREFVGTFGGIAFSLAATYFAFQAGAGAL-ININQQI----------  -------GPSE--NYYLF------SIVWTVS-----QPISMLLFGRLSDMFGRRNLALGA  NILGIIGGIIAA--TSQSINQLIAANVLLGLASGIPGSYPLLTGELMTNKLKFLGTIVVV  VPNIIATGFGPYLGLRLSIL-SSWRWIFYIYIILMVIGTTLWYFFYHPPSFVQLHGTSIS  RKAELATIDWLGTFLLVSGLVLFLLGIAWGGQP--YKWTSGRVLGLLIPGAILSIAFILY  EVYGKPAKPIVPMKFF-KDVRGYTCVVIISAITGCLQTALFILWPSQVAYIF--GSTTNG  WEETAWMSSVVN----FAAWAGIIIVGPLFHVIKHLRLQLVVGSAWMTAFLAAMSTITYS  NKGSAIAFAFLSTLPIGWGEVMTMLMVQYIVPERDLGVAF------AVVSSVRTIMGSIF  AAVFVAIYT-----------------NKLPGYMTSIVIPAL-QKTSLSAEAISEVLA--T  APTASQAAL-SAIKGV--TPEILRVINAKVADAYGHSYAFVYYTAAALGAVCLLAALCLR  DFDVYLTDHVARMVYKKEETKEDSLAK----HIEHPEGV---------------------  ---------------  >FproTRI12b  ----------------------MTKQEASPDDISPGHNNPDTPL-SNEDELRSKILTAER  S----TLPEGYFLSREFVGTFGGIAFSLAATYFAFQAGAGAL-ININQQI----------  -------GPSE--NYYLF------SIVWTVS-----QPISMLLFGRLSDMFGRRNLALGA  NILGIIGGIIAA--TSQSINQLIAANVLLGLASGIPGSYPLLTGELMTNKLKFLGTIVVV  VPNIIATGFGPYLGLRLSIL-SSWRWIFYIYIILMVIGTTLWYFFYHPPSFVQLHGTSIS  RKAELATIDWLGTFLLVSGLVLFLLGIAWGGQP--YKWTSGRVLGLLIPGAILSIAFVLY  EVYGKPAKPIVPMKFF-KDVRGYTCVVIISAITGCLQTALFILWPSQVAYIF--GSTTNG  WEETAWMSSVVN----FAAWAGIIIVGPLFHVIKHLRLQLVVGSAWMTAFLAAMSTITYS  NKGSAIAFAFLSTFPIGWGEVMTMLMVQYIVPERDLGVAF------AVVSSVRTIMGSIF  AAVFVAIYT-----------------NKLPGYMTSIVVPAL-QKTSLSAEAISEVLA--T  APTASQAAL-SAIKGV--TPEILRVINAKVADAYGHSYAFVYYTAAALGAVCLLAALCLR  DFDVYLTDHVARMVYKKEETKEDX------------------------------------  ---------------  >FoxyTRI12b  ----------------------MTKQEASQDDISPGHNDQDIAL-SNEDELRSKILTAER  S----TLPDGYFLSREFVGTFGGIAFSLAATYFAFQAGAGAL-ININQHI----------  -------GPSE--NYYLF------SIVWTVS-----QPISMLLFGRLSDMFGRRNLALGA  NVLGICGGIVAA--TSQSINQLIAANVLLGLASGIPGSYPLLTGELMTNKLKFLGTIVVV  VPNIIATGFGPYLGLRLSIL-SSWRWIFYIYIILMVIGTTLWYFFYHPPSFVQLHGTSIS  RKAELATIDWVGTFLLVSGLVLFLLGIAWGGQP--YKWTSGRVLGLLIPGAVCSIAFVLY  EIYGQPAKPIVPMKFF-KDVRGYTCVVIISAITGCLQTALFILWPSQVAYIF--GSTTNG  WEETAWMSSVVN----FAAWAGIIIVGPLFHVIKHLRLQLVVGSAWMTAFLAAMSTITYS  NKGSAIAFAFLSTFPIGWGEVMTMLMVQYIVPERDLGVAF------AVVSSVRTIMGSIF  AAVFVAIYT-----------------NKLPGYMASIVVPAL-QKTSLSADAISEVLA--T  APTANQAAL-SAIKGV--TPEILRVINAKVADAYGHSYAFVYYTAAALGAVCFFAAMCLR  DFDVYLTDHVARMVYKKEETKEDSLAK----XIEX-------------------------  ---------------  >FOPG_13450  ----------------------MTKQEASQDDISPGHNDQDVAL-SNEDELRSKILTAER  S----TLPDGYFLSREFVGTFGGIAFSLAATYFAFQAGAGAL-ININQHI----------  -------GPSE--NYYLF------SIVWTVS-----QPISMLLFGRLSDMFGRRNLALGA  NVLGICGGIVAA--TSQSINQLIAANVLLGLASGIPGSYPLLTGELMTNKLKFLGTIVVV  VPNIIATGFGPYLGLRLSIL-SSWRWIFYIYIILMVIGTTLWYFFYHPPSFVQLHGTSIS  RKAELATIDWVGTFLLVSGLVPFLLGIAWGGQP--YKWTSGRVLGLLIPGAVCSIAFVLY  EIYGQPAKPIVPMKFF-KDVRGYTCVVIISAITGCLQTALFILWPSQVAYIF--GSTTNG  WEETAWMSSVVN----FAAWAGIIIVGPLFHVIKHLRLQLVVGSAWMTAFLAAMSTITYS  NKGSAIAFAFLSTFPIGWGEVMTMLMVQYIVPERDLGVAF------AVVSSVRTIMGSIF  AAVFVAIYT-----------------NKLPGYMASIVVPAL-QKTSLSADAISEVLA--T  APTANQAAL-SAIKGV--TPEILRVINAKVADAYGHSYAFVYYTAAALGAVCFFAAMCLR  DFDVYLTDHVARMVYKKEETKEDSLAK----HIEHREGA---------------------  ---------------  >UCRNP2_10378  MEEMN--------------NLPVPDKTT--------INVALLSQ-DEADKLRSQALSSDR  E----KIPAGYYSSARIVGTFVGIALSLVGTYFVYQAAASVI-TSINEDI----------  -------GPSE--NSSLF------AIVWTAA-----QPISILLFGSLSDRFGRRKWALAA  NVLGILGGIVAG--TAKTMNTLIGAMVMLGLASGVPASYPLLTGELATNKGKFLATITVV  VPNVIATGFGPYIGQRLVVY-ATWRWIFWIYIMMMVPGTVCWYFWYHPPTFVQLHGHNFE  RRQALKRIDYFGVLLLTAGLTLFLLGVSWGGTTPALTWDSARILGLLIPGIVCCVAFVIY  ECL-VPESPIIPMRFF-RDVRGFACINVISAVFGCINIAFFILWPSQVIHIF--GST-GS  WEQTAWMSCTVN----FGIWAGIIIVGPLYHIIKHIRWQLVVATVWMSVFLGVITTANAN  QRAAAIAVSFLSCLPIGWGEVITMLLVQYLVDDRDLGAAF------AVTSANRTILGCIF  TSVFSAILT-----------------NKLPQELSAHLVP--------------------P  VVAGNSAAL-SLVPGLD-DPTVLKAVNEGASSAWAGAYA---------------------  DMDKYLTGHIPRQIYTKTA-----------------------------------------  ---------------  >UCDDS831_g00713  MEEKS--------------SPPVPEKKT--------INVALLSQ-DEADKLRSQALSSDR  D----TIPPGYFSSIRIVGTFVGIALALVATYFAYQAGAAVI-ISINEDI----------  -------GPSE--NSSLF------AIVWTAA-----QPISILLFGSLSDRFGRRNWALAA  NVLGIVGAIVAC--TAKTMNTLIGAMVLLGLASGVPASYPLLTGELATNKGKFLATITVV  VPNVIATGFGPYIGQRLVVY-SSWRWIFIIYIIMMVPATVCWYLWYHPPTFVQLHGHNFK  RTEALKHIDYLGVLLLTAGLTLFLLGVSWGGTTTALTWDSARILGLLISGIICCIAFVIY  ECL-VPASPIVPMRFF-KDVRGFACINVMSAVFGCINIAFFILWPSQVIHIF--GST-GS  WQETAWMSCTVN----FGIWAGIIIVGPLYHIIKHIRWQLVVATAWMSVFLGVITTFNSG  QRPAAIAVSFLSCLPIGWGEVVTMLLVQYLVDDRDLGAAF------AVTSANRTILGCIF  TSVFSAILT-----------------NKLPQQLAAVLVPRA-LQAGLDPSAVPAVLA--A  VASANATAL-AQVPALRADPHLLAVVNKGASDSWAGAYAQIY------------------  ---ERGEGGV------------DLLERE---DVAAQLDG------GRREA----------  ---------------  >UCREL1_6328  M------------------TASQATESTVAPP-------SPPTTTDNVDKLRSEALAADS  K----QLPQGYYSSTRVIGTFGGIGFSLLGTYWAFSVGAAVI-TAINQDI----------  -------GPSA--NSSLF------SIVWTTC-----NCISILLFGRLSDRFGRRGLAISS  NLLGIIGGIVAA--TAPTMNALIGANVLIGLSSGPSASYPLLTGELATNKTKYLATVLVV  IPNVIATGFGAYLGQRIVLQ-TTWRWIFIIYIIFMVPGTVLYYLFYYPPSFTQMHGKDKK  AIEEVKKIDFAGLFLLVAGLALFLLGVSWG------------------------------  ---------------------GFTPLVIISAVTGCLNVALRITWPSQVLKVF--GT--SD  WETAAWLTTTVA----FGSWAGIVIFGSFYHVIKRIRWQLVVGCVWMSAFVGAMASIDPT  ELNQAAAFSFLATLPVGWGEVITMVMVH--------------------------IVGAIF  TAVFTA------------------------------------------------------  -------EC-RAVPGM--NDGILAVTSTKVSEAYAAAYAYPYYTALALALVSVIAAIGIR  DFDHYLTDHVSRQIYKKDDTGKDILQLVNYPDTEQRGIVTELKS-TEKDS----------  -------NDLPAQ-V  >PV10_06067  MADSGLRHRGH--------HDHTPDELTELQRLELEH--AQRDAGALEDLQNSDALVTDQ  S----KFTGRYYF--RMATACFSISMGTTGSYFGFTCPASLL-TYINEDI----------  -------GPSN--NASLV------SIIWTAA-----CAIAIILFGRLSDKFGRRWFVVGA  SFMGVIGGIIAS--RAQNMDTLVGANVLIGLSGGVHTCYGLTMGELVPNRYKTAAFSAVV  VLCFVGISFAPIIGFRLIKLGPGWRFIYYIYIIFMAIATISQFFFYKPPSFKQLHGGKRT  IMQEVKRIDFVGLFLLTAGMVLFILGISWGGQP--VPWKSAQILCLLIIGFVLCVLFVLW  EIYSNVPNPLMPMYFF-KDVRGFTCLAIIDAISGAGYVAPSVIWPMQVANIYAAGSNPVG  WEEQAWLTSAVT----FAIVGGIVFWGAILPIIKHVKKQMILMALMVLAFSGGLVASSRD  NKGRNAAFAFLSMFADGMLEITPLSLVQISANDADLGTVF------GIICLFRTVTGSIF  TAIYLAIFQ-----------------NTLPEEIAKFVPSAV-EAAGLPSSSLDELMT--A  VGDGTADAL-NAVPGM--TGGILNATLDALVDAKTASFSYMYYATIPVNVIMLGAVLLMK  DYDKLLTTHVPRQVYANGEGVEHHEKFG---DVEGSPAHPS----SEKVQNLEIEAAAAG  SNSDR--EDVR----  >PV07_12085  ------------------------------------------------------------  ------------------------------------------------------------  ------------------------------------------------------------  ------------------MDTLVAANVFLGLSGGVHTCYALTVGEICPNKFKLAGSAFVT  IPCILSTGFGAYISSRLAHTGAGWRWCYYSYLMMMSCAAIMQYHFYKPPSFKQLHGGRRT  LKEEFRRIDFVGGFLLTAGLVMFLLGISWGGQP--IPWTSPRILCLIVIGAVLIVAFVFW  EIYSTTPNPLVPMYFF-RDLRGFTCLVIICSVSGASYVAPTVVWPSQVAAVY--GVRTTN  WQENAWLNTTVS----FGIIGGIWFWGPMVSVVKHVRIQLLVLATMTCAFNGALASSNRD  NKQQSAAFSFLATLPDGIMELMPVALAQMDANDADLGTVF------GIVFLFRTVCGSIF  TTIYLAILQ-----------------NKLLFAIAQRVPAAA-EAAGLPASSLKDVLT--A  ASVGTAAAL-DKVPGM--NPAIATQIMDALVEARVWSYKFIYLASIPINFCAVVAAFFIK  DYDPLLSSHVPRQIYAHGEGVKKSQEKGT-IDIEDSSGERD----GEKSQTVEIDDAAAA  PAHPA--SDMRETKN  >PV07_00317  MSKPTPFETPWREPPAACPPVFLKEAITNLQRLELEH--AQRDAGTIADYQNSRALTTDQ  S----KFNATYLL--RLVSAVAGISLGTVAAYWGFSPPAAVL-TYISEDL----------  -------GDAE--NASLF------SIVWTAA-----CAVSIILFGRLSDKFGRRYFMIGA  SVIGVIGGIIAC--TAKTMNVLIGANVLLGLSGGVHTCYGLTVGEICPNRYKFLGITFCV  IPSILTTGFGAYISLRLVNTGAGWRWCYYTYLMMMGAAAALQIFFYHPPSFQQLHGGKRT  VMEEVKRIDFVGIFLMTAGLSMFLLGVSWGGSP--LPWTSARILSLLIVGGVTLVVFVFY  EIYSGVANPLIPMYFF-KDLRGFTCLVVICGISGISYVAPSIIWPTQVAEIY--GLNTTG  WQENAWLSTTIA----FGIIGGIYLWGPMIAVVKHVKWQTVVLAAMVMAFSGALASSNQY  NRGQSAAFSFLTTLPGGILELIPVSLVQMEANDADLGTVF------AILFLARTTLGSIF  ATIYLAILN-----------------NKLPGEIASKVPQAA-LAAGLPKSSLADLLQ--A  AAAGTTAAF-SAVPGM--TPEIQQAAINALLHARSVAYSYIYYSSIPVNFCGVVAALCMR  DYDHLLNSHVPRQIYSHGQGADAAEKQAA--DEERSPVETVDAVKNEKKPWRQVESTSSE  ---------------  >UCRPC4_g01346  MD--------------------APVAETKLKQLELSH--ANED--TLADIKNSDALATEQ  Q----ELEAGYFLSARLVFSVLGVSLSTLATYWGFSPAAAVL-TTINADI----------  -------GPTD--NDTLF------SIIWSTC-----SAISVILFGRISDKFGRRWFLIGA  SVMGFIGGIIAC--TAQTMTTLVGANVLLGLGAGVHTCYSLTVGEICPNKYKFLGVAFCV  LPSVIPTGFGAYLGAMLVHT-ANWRWIYYIYIIVMGVSVVLQVLFYRPPSFRQLHGNSRT  VMQEVKRIDFVGCLLLVAGLCLFLLGISWGGQP--LPWTSSRILGLVISGGVLLVVFVLW  EIYSQHPNPLVPMHFF-KDVRGFLCLNVIAAVSGTTYIALSILWPSQVNAIY--SARATS  WQSTAWLSTTIA----FGIWGGIVTLGPLVGVVKHVRYMTTILMAVSVAFLGGLASCNSS  NFGQSAAFSFLATYPAGILELIPGILVQLDSNDADLGTAF------SIVFLIRAAFGTIM  TAVFVAILS-----------------AKATPEIISHVTPAA-LEAGLPQSSLTDLFT--A  IAAGTTAAL-EAVPGM--TTEIEAAVADALSDGYAAAYSYVYYAAVAVGIVGLIACISLK  DYDSLFTGHVSRQIYKPSDDIKSVDEST---EAEKSDVEKA----SVKEPVAQETSVS--  ---------IRNE--  >PV07_06272  MD----------------------------------------------------------  -----LLKGG---TAVIIGAASGIGQAAAIAFARDGCKKVMI-GDINVD-----------  -------GLAE--RKRLIQEQYPDTVVETAAIDVVDESSVEAFHQEAASRFGRIDF--AA  NSAGYGDGIVAC--RAQTMNTLIGANVMLGLSAGVHTCYALIIGEICSHKHKFIGVVLVL  VPNVVATGFGAYIALDLAHN-ASWRWVYYIFIMMMSASIALQYVFYRPPNFRQLHGDDRT  RWEEVKRIDWVGLFFLVAGLVLFLLGISWGGQP--LPWSSTRVLGLVISGGITLIVFGLW  EGFSNTTNPLVPMHFF-KDLRGFVVLCLISSVCGTVYLATAIIWPSQVAVIY--SGTITS  WQHIAWASLTNANYHCVRCLVGMIFIGPFVGIIKHVRIQFIVLMSICVAFLGGLVSCNPH  NFGQSAAFFFLAAMPLGIMEMTARLLVQMDSNDADLGTVF------SIVFLIKSASGSIF  TSVFIAILN-----------------NKLPAELKKYVIPAA-LQAGLPQSSLTALAG--A  VTSGNSTLL-THVPGM--DTAIEAAVGSGLATAYAAAYAYVYYAAVALP----------K  NYDPLFNDHISRKIYKSGQNDAPTQ-----------------------------------  ---------------  >PABG_01811  M------------------SVKDKVDSTEREVAMPQAPAAASTGNP-SSHDLAEELALEA  THHVEQVGWKFLMEPYTVGSLAAMSFGLFNAAWGFAPPAAVL-LWINNDIGMGPIETVVF  VHILTVLGSNSKTNAALF------SVMQTLG-----TTLGYMFVGRLSEIYGRRWVMIIF  TLFGLAGSIIAG--TSQDLNTFVGANVLLGLSTGAQCCYAFLAGELMPNKHKMLGMAIVV  IFCLPGTSLGAYLARSLVEQ-ASWRWIYYIYIIAQTISLALYYFFYFPREAAQAY----N  KIEQTKKLDFVGIFLLLAGLVLFIVGIMTGGSP--YPWKSAKIIGMVVSGGISLIALCVW  EHF-QGENAFFAVHLF-KDVWGFGMNCICSAVGGISYTALSIIWPTQVAYMY---SAGSS  WETVAAISCTVG----FGLWGGMVFLGPLWGPIGHPKMTLIVSKMWMVAFTAALANCNPD  NKKFAIACSFLAALPIGFVEQQTGAIAQLVVSDKHIGTSF------GTMGCVRVGVGVIG  TAIVLAILS-----------------AKIPTELQAHVVPAA-LNAGLPKSSIADLFA--A  LTKATAEAM-AAVPGI--NSEIIAAVSRAQLEGYAAAYRYIYFATIPFGVVATASAIALR  PIKHLLTNHVPKIVEHPQTHL----GKD---DLEKSAA----------------------  ---------------  >PAAG_01242  M------------------SVKDKVDSTELEVAMPQAPAAASTGTP-ASHDLAEELALEA  THHVEQVGWKFLMEPYTVGSLAAMSFGLFNAAWGFAPPAAVL-LWINNDI----------  -------GSNSKTNAALF------SVMQTLG-----TTLGYMFVGRLSEIYGRRWVMIIF  TLFGLVGSIVAG--TAQDLNTFVGANVLLGLSTGAQCCYAFLAGELMPNKHKMLGMAIVV  IFCLPGTSLGAYLARSLVEH-ASWRWIYYIYIIAQTISLALYYFFYFPREAAQAY----N  KLEQTKKLDFVGIFLLLAGLVLFIVGIMTGGSP--YPWKSAKIIGMVVSGGISLIALCVW  EHF-QGENAFFAVHLF-KDVWGFGMNCICSAVGGISYTALSIIWPTQVAYMY---SAGST  WETVAAISCTVG----FGLWGGMVFLGPLWGPIGHPKMTLIVSKMWMVAFTAALAHCNPD  NKKFAIACSFLAALPIGFVEQQTGAIAQLVVSDKHIGTSF------GTMGCVRVGVGVIG  TAIVLAILS-----------------AKIPVELQAHVVPAA-LNAGLPESSLADLFA--A  LAKATAEAM-ASVPGI--NSEIIAAVTRAQLEGYAAAYRYIYYTTIPFGVVATASAIALR  PIKYLLTNHVPKIVEHPQAHL----GKD---DLEKSAA----------------------  ---------------  >EMCG_06273  M------------------SIKDKGGSTEVEVAMTQPPAPISTGTP-DSHDLAEELALEA  TCYVEQVGWKFLMEPYTAGSLAAMSVGLFNAAWGFAPPAAVL-LWINADI----------  -------GSNSQTNGALF------SVMQTLG-----TTLGYMFVGRLSEIYGRRWVMIIF  TLFGLVGSIIAG--TAQDLNTFIGANVLLGLATGAQCCYAFLAGELMPNKHKMLGMAIVV  IFCLPGTSMGAYLARSLVEH-ASWRWIYYIYIIAQTISLALYYFFYFPREAAQSY----N  KLEQTKKLDFVGIFLLLAGLVLFIVGIMTGGSP--YPWQSAKVIGMVVSGGISLVALCVW  EHF-QGENAFFAVYLF-KDVRGFGMNCICSAVGGISYTALSIIWPTQVAYMY---SAGSS  WETVAAMSCTVG----FGLWAGMVFLGPLWGPIGHPKMTLIVSKMWMVAFTAALAHCNPD  NKKFAIACSFLAALPIGFVEQQTGAIAQLVVSDKHIGTSF------GTMGCVRVGVGVIG  TAIVLAILA-----------------AKIPIELEAHVVPAA-LNAGLPEGSLADLFA--A  LAKTTVEAM-AAVPGI--NSEIIAAVTRAQLEGYAAAYRYIYYTTIPFGVVATASAMALR  PIKHLLTNHVPKIVGSPHAQL----GKD---DLEKSVV----------------------  ---------------  >EMPG_09549  M------------------SDNDKGESTETEVAKTGLPVSAPAG-PLASQDLVEELALEA  TKHVEAVGWKFLMERNTLGSLAAMSFGLFNAAWGFAPPAAVL-LWINADI----------  -------GSESQTNGALF------SVMQTLG-----TTLGYMFVGRLSEIYGRRWVMLMF  TLFGLIGSIVAG--TANDLNTFIGANILLGLATGAQCCYAFLAGELMPNKHKMVGMAIVV  IFCFPGTSMGAYLARSLVEH-ASWRWIYYIYIIAQIISLALYYFFYFPREAAQAY----N  KLEQTKKLDFVGIFFLLAGLVLFIVGIMTGGSP--YPWKSAKVIGMVASGGISLIALCVW  ESF-QGENAFFAVHLF-KDVWGFGMNCIISAVGGISYTALSIIWPTQVSYMY---SAGSS  WQDVAAMSCTIG----FGLWGGMVFLGPLWGPIGHPKMTLIVSKIWMVVFTAALANCNPD  NKKFAIACSFLAALPIGFVEQQTGAIAQLVVPDKHIGTSF------GTMGCVRVGVGVIG  TAIVLAILA-----------------AKIPVELQNHVVPAA-LGAGLPETSIPDLFA--A  MAKATQDAM-AAVPGI--NPAIIAAVGRAQQEGYAAAYRYIYYTTIPFGVVATAAAIALR  PIKHLLTSHVPKIVEHPQQTLVNVVGKG---DIEKAGA----------------------  ---------------  >V499_03270  M---------------------------TIENITPQHLESQLTATPDNVELLAEELAVEA  THHVDAVGWKFLIEPYTFGSLAAMSFGLFCAAWGFAPPASVL-IWITADI----------  -------GSKSQTNAALF------SVMQTIG-----TTLGYMFVGRLSEIYGRRWVMIVF  TLFGLVGSIVAG--TSHDLNTFIGANILMGLATGAQCCYAFLAGELMPNKHKMLGMAIVV  LFCLPGTAMGAFFARSLVEY-ASWRWIYYIYIIAQTISLALYIVCYFPKEAAQAF----N  KREQTKKLDFIGMFLLVAGLVLFILGIMTGGSP--YPWKSAKVLGMVVSGGAA-------  ----------------------------------------------EVAYMY--STGGSS  WQAIGAMSCTIG----FGLWGGMVFLGALWGPIGHPKWTLIISNIWMTAFIGALAHCNPD  NKTFAIVCSFLAALPIGFIEQQTGAIAQLVVGDKEIGTSF------GTMGCVRVGTGAIG  TGIVLAILTGEHFKYIYERPETDIFKAKIPVELEAHIVPAA-LNAGLPASSIADLFA--A  IFASSETAM-AAVPGI--NAEIIKAVGKAQLDGYAAAYRYIYYATIPFGVLATASAVALR  PVAHLLTSHVPKMVENPQAYH----GNR---DLEKTMD----------------------  ---------------  >V501_03398  M---------------------------TIENISPQHLESQLTATPHNVELLAEELALEA  THYVDAVGWKFLIEPYTFGSLAAMSFGLFCAAWGFAPPASVL-IWINADI----------  -------GSKSQTNGALF------SVMQTIG-----TTLGYMFVGRLSEIYGRRWVMIVF  TLFGLVGSIVAG--TSHDLNTFIGANILMGLATGAQCCYAFLAGELMPNKHKMLGMAIVV  LFCLPGTAMGAFFARSLVEY-ASWRWIYYIYIIAQTISLALYIVCYFPKEAAQAF----N  KREQTKKLDFIGMFLLVAGLVLFILGIMTGGSP--YPWKSAKVLGMVVSGGVFLIALCVW  EVY-QGEHAFFAIHLF-KDVRGFGMNCICSAVGGISYVALSIIWPTR-------------  -------------------------------PIGHPKWTLIISNIWMTAFIGALAHCNPD  NKIFAIVCSFLAALPIGFIEQQTGAIAQLVVGDKEIGTSF------GTMGCVRVGTGAIG  TGIVLAILTGEHFKYIYERPETDIFKAKIPVELEAHIVPAA-LNAGLPASSIADLFA--A  IFASSETAM-AAVPGI--NAEIIKAVDRAQLDGYAAAYRYIYYATIPFGVLATASAIALR  PVAHLLTSHVPKMVENPQAHH----GNR---DLEKTMD----------------------  ---------------  >V490_02382  MTLQN--------------NEGNMQPAAEQDTISPQHPVSELTATPFNTALVAEEIALEA  THHVDAVGWKFLIEPNTFGSLAAMSFGLFCAAWGFAPPASVL-LWINADI----------  -------GSESQGNGALF------SVMQTIG-----TTLGYMFVGRLSEIYGRRWVMIAF  TLFGLVGSIIAG--TAQNLNTFIGANILMGLATGAQCCYAFLAGELMPNKHKMLGMAIVV  LFCFPGTAIGALLARSLVEH-VSWRWIYYIYIIAQTISLALYVIFYFPKEAARAF----N  KREQTKKLDFVGIFLLVAGLVLFILGIMTGGSP--YPWKSAKVLGMVVSGGISLIALCVW  ESF-QGENAFFAVHLF-KDVRGFGMNCICSAVGGISYVALSIIWPTQVAYMY---STGSS  WQAVGALSCTIG----IGLWAGMVFLGALWGPIGHPKLTLIISNMWMTAFIGALAHCNPD  SSAFAAACSFLAALPIGFIEQQTGAISQLVVGDKDIGTSF------GTMGCVRVGVGAIG  TGIVLAILT-----------------SKLPVELEAHIVPAA-LGAGLPASSLADLFS--A  LLVASETAL-AAVPGI--NNEIIAAVLRAQLDGYAAAYRYIYYTTIPFGVLATASAVALR  PVAHLLTDHVPKMVESAQAHH----GEK---DLKKSMV----------------------  ---------------  >O988_01318  MTLQN--------------NEVNMEPATEPNTISPQHPASELTATPFNTALIAEEIALEA  THHVDAVGWKFLIEPNTFGSLAAMSFGLFCDAWGFAPPASVL-LWINADI----------  -------GSESQSNGALF------SVMQTIG-----TTLGYMFVGRLSEIYGRRWVMIAF  TLFGLVGSIIAG--TAQNLNTFIGANILMGLATGAQCCYAFLAGELMPNKHKMLGMAIVV  LFCFPGTAIGALLARSLVEH-VSWRWIYYIYIIAQAISLALYVTFYFPKEAARAF----N  KREQTKKLDFVGIFLLVAGLVLFILGIMTGGSP--YPWKSAKVLGMVVSGGVSLIALCVW  ESF-QGENAFFAVHLF-KDVRGFGMNCICSAVGGISYVALSIIWPTQVAYMY---STGSS  WQAVGALSCTIG----IGLWAGMVFLGALWGPIGHPKVTLIISNMWMTAFIGALAHCNPD  SRAFAAACSFLAALPIGFIEQQTGAISQLVVGDKDIGTSF------GTMGCVRVGVGAIG  TGIVLAILT-----------------AKLPVELEAHIVPAA-LNAGLPASSLADLFS--A  LLAASEAAL-AAVPGI--NNEIISAVLRAQLDGYAAAYRYIYYTTIPFGVLATASAIALR  PVAHLLTDHVPKMVESAQAHH----GEK---DLKKSMV----------------------  ---------------  >V500_04545  MALEN--------------RAGDMEPTMELEHISPQHTVSKSTATPFNAELVAEEISLEA  THYVEAVGWKFLIEPNTFGSLAAMSFGLFCAAWGFAPPASVL-LWINADI----------  -------GSESQTNGALF------SVMQTIG-----TTLGYMFVGRLSEIYGRRWVMIVF  TLFGLVGAIIAG--TAQNLNTFIGANILLGLATGAQCCYAFLAGELMPNKHKMLGMAIVV  LFCFPGTAIGALLARSLVEH-VSWRWIYYIYIIAQTISLALYVIFYFPKEAAQAF----N  KREQTKKLDFVGIFLLVAGLVLFILGIMTGGSP--YPWKSAKVLGMVVSGGVCLIALSVW  ESY-QGENAFFAVHLF-KDVRGFGMNCICSAVGGISYVALSIIWPTQVAYMY---STGSS  WQSVGALSCTIG----IGLWGGMVFLGSLWGPIGHPKLTLIFSNVWMTIFIGALAHCNPD  NRTFAAACSFLAALPIGFIEQQTGAIAQLVVGDKDIGTSF------GTMGCVRVGVGAIG  TGIVLAILT-----------------AKLPVELEAHIVPAA-LNAGLPASSLADLFA--A  LLSASETAL-AAVPGI--NSEIISAVGRAQLGGYAAAYRYIYYATIPFGVLATASAIALR  PVAHLLTDHVPKRVENAQAHH----GTR---DLKNTMV----------------------  --------------- |
| --- |

**Supplementary Data 3.** Full alignment of nucleotide sequences (NEXUS format) of *Tri12* gene fragments used to construct maximum likelihood phylogeny of *F. oxysporum* and *F. proliferatum* isolates, depicted on Figure 3 of the main manuscript. Includes *FsTri12* outgroup.

| #NEXUS  [saved by seaview on Thu Nov 5 14:10:06 2015]  BEGIN DATA;  DIMENSIONS NTAX=27 NCHAR=1851;  FORMAT DATATYPE=DNA  GAP=-  ;  MATRIX  [1] 21L_Fp  atgacgaaacaagaagcatccccagatgacatctcgccaggtcacaataacccagacact  cccctctcaaacgaagatgaacttcgctccaagatcttaaccgctgagcgttccactctc  ccggaaggatatttcctcagccgggagttcgtcggaacgtttggcggtatcgcgttcagt  cttgcggcgacatactttgcctttcaggctggtgccggtgccctaatcaacatcaaccag  cagatcggcccaagtgagaactactacctgttctcaatcgtatggacggtctctcagccc  atctctatgctcttgtttggtcgtctctctgatatgtttggtcgccgaaaccttgctctt  ggtgccaacattcttggaataatcggcggtatcattgctgccacttcacagagcatcaac  caactcattgctgcaaatgttcttttgggtcttgcctctggcattccgggttcttaccct  cttttgacgggcgagcttatgacaaataaactcaagtttcttggtactatcgttgttgtg  gtgcccaacatcatcgccactgggttcggtccgtatcttggccttcgacttagtattctt  agttcatggagatggatcttttacatctacatcatcttgatgggtatgcctccaagcctc  acgcaacgcaaggatgacaagaaccacactaacaa---cttgtagtaatcggtactaccc  tctggtacttcttctaccaccctccttcgtttgtccaactccacggaaccagcatcagcc  gcaaagccgagcttgcaacaatcgactggcttggcacttttcttcttgtctcaggtctcg  tccttttccttcttggaatcgcctggggaggacaaccatacaa---atggacatccggtc  gagtccttggtcttctgatccctggtgcagttttgtccatctcattcgttctctatgacg  tatacggcaaccctgcaaagccaatcgtgcccatgaagttcttcaaggatgtccgaggct  acacatgtgtcgtcatcatctctgccattaccggctgcctccaaacggccctgttcatct  tgtggccttctcaagtcgcatacatcttcggaagcacgactaatggatgggaggagactg  cttggatgtcgagcgttgtcaactttgctgcttgggccggtatcatcattgttgggcctc  tcttccacgtcatcaaacatttgcgtctacagcttgttgtcggatctgcctggatgacgg  ctttcctggcggccatgtctactatcacctactccaacaagggatctgctattgccttcg  cctttctcagcacctttcccatcggctggggagaggtcatgactatgctcatggttcagt  acattgtcccggaacgagatctgggtgttgcgtttggtaagtagccaggcctgtccaagt  ccctactgt-ttcatgactaaccgcaatttcctgatagccgtcgtctcctcagtccgtac  catcatgggctccatcttcgccgctgtcttcgtcgccatctacaccaacaagctcccagg  ctacatgaccagcattgtagtaccagcgctacaaaagacgtctctttctgcagaggctat  tagtgaagtactggcaacagcacctactgcgagccaggccgcactctccgctatcaaagg  tgtcactcctgagatcttgcgtgtcatcaacgccaaggtcgcagatgcttacggtcactc  atatgcctttgtttactacaccgctgctgctctcggggctgtttgccttcttgcggcatt  gtgtctgcgtgacttcgatgtttacctgactgaccacgttgcccgcatg--  [2] 59L_Fp  atgacgaaacaagaagcatccccagatgacatctcgccaggtcacaataacccagacact  cccctctcaaacgaagatgaacttcgctccaagatcttaaccgctgagcgttccactctc  ccagaaggatatttcctcagccgggagttcgtcggaacgtttggcggtatcgcgttcagt  cttgcggcgacatactttgccttccaggctggtgccggtgccctaatcaacatcaaccag  cagatcggcccaagcgagaactactacctgttctcaatcgtatggacggtctctcagccc  atctctatgctcttgtttggtcgtctctctgatatgtttggtcgccgaaaccttgctctt  ggtgccaacattcttggaataatcggcggtatcattgctgccacttcgcagagcatcaac  caactcattgctgcaaatgttcttttgggtcttgcctctggcattccgggttcttaccct  cttttgacgggcgagcttatgacaaataaactcaaatttcttggtactatcgttgttgtg  gtgcccaacatcatcgccactgggttcggtccgtatcttggccttcgacttagtattctt  agttcatggagatggatcttttacatctacatcatcttgatgggtatgcctccaagcctc  acgcaacgcaaggatgacaagaaccacactaacaa---cttgtagtaatcggtactaccc  tctggtacttcttctaccaccctccttcgtttgtccaactccacggaaccagcatcagcc  gcaaagccgagcttgcaacgatcgactggcttggcacttttcttcttgtctcaggtctcg  tccttttccttcttgggatcgcctggggaggacaaccatacaa---atggacatccggtc  gagtccttggtcttctgatccctggtgcagttttgtccatctcattcgttctctatgagg  tatacggcaaccctgcaaagccaatcgtgcccatgaagttcttcaaggatgtccgaggct  acacatgtgtcgtcatcatctctgccattaccggctgcctccaaacggccctgttcatct  tgtggccttctcaagtcgcatacatcttcggaagcacgactaatggatgggaggagactg  cttggatgtcgagcgttgtcaactttgctgcttgggccggtatcatcattgttgggcctc  tcttccacgtcatcaaacatttgcgtctacagcttgttgtcggatctgcctggatgacgg  ctttcctggcggccatgtctactatcacctactccaacaagggatctgctattgccttcg  cctttctcagcacctttcccatcggctggggagaggtcatgactatgctcatggttcagt  acattgtcccggaacgagatctgggtgttgcgtttggtaagtagccaggcctgtccaagt  ccctactgt-ttcatgactaaccgcaatttcctgatagccgtcgtctcctcagtccgtac  catcatgggctccatcttcgccgctgtcttcgtcgccatctacaccaacaagctcccagg  ctacatgaccagcattgtagtaccagcgctgcaaaaggcgtctctttctgcagaggctat  cagtgaagtactggcaacagcacctactgcgagccaggccgcactctccgctatcaaagg  tgtcactcctgagatcttgcgtgtcatcaacgccaaggtcgcagatgcttacggtcactc  atatgcctttgtttactacaccgctgctgctctcggggctgtttgccttcttgcggcatt  gtgtctgcgtgactttgatgtttacctgactgatcacgttgcccgcatg--  [3] 66L_Fp  atggcgaaacaagaagcatccccagatgacatctcgccaggtcacaataacccagacact  cccctctcaaacgaagatgaacttcgctccaagatcttaaccgctgagcgttccactctc  ccagaaggatatttcctcagccgggagttcgtcggaacgtttggcggtatcgcgttcagt  cttgcggcgacatactttgcctttcaggctggtgccggtgccctaatcaacatcaaccag  cagatcggcccaagtgagaactactacctgttctcaatcgtatggacggtctctcagccc  atctctatgctcttgtttggtcgtctctctgatatgtttggtcgccgaaaccttgctctt  ggtgccaacattcttggaataatcggcggtatcattgctgccacttcgcagagcatcaac  caactcattgcagcaaatgttcttttgggtcttgcctctggcattccgggttcttaccct  cttttgaccggcgagcttatgacgaacaagctcaagtttcttggtactatcgttgttgtg  gtgcccaacatcatcgccactgggttcggtccgtatcttggccttcgacttagtattctt  agttcatggagatggatcttttacatctacatcatcttgatgggtatgcctccaagcctc  acgcaacgcaaggatgacaagaaccacgctaacaa---cttgtagtaatcggtactaccc  tctggtacttcttctaccatcctccttcgtttgtccaacttcacggaaccagcatcagcc  gcaaggccgagcttgcaaccatcgactggcttggcacttttcttcttgtctcgggtctcg  ttctcttccttcttgggatcgcctggggagggcaaccatacaa---atggacatcaggtc  gcgtccttggtcttctgatccctggtgcaattttgtccatagcattcgttctctatgagg  tatacggcaagcctgccaaaccaatcgtacccatgaagttcttcaaggatgcccgaggct  acacatgcgtcgtcatcatttccgcaatcactggctgccttcagacggccttgttcatct  tgtggccttctcaagtcgcatacatcttcggaagcaccaccaatggatgggaggagactg  cttggatgtcaagcgttgtgaactttgcagcttgggctggtatcatcgtcgtcgggcctc  tcttccacgtcatcaagcatttacgtctacagcttgttgtcggatctgcctggatgacag  ctttcctggcggccatgtcgaccatcacctactccaacaagggatcggctattgctttcg  cctttctcagcacctttcccatcgggtggggagaggtcatgactatgttgatggttcagt  acattgttccggaacgagatctaggtgttgcctttggtaagtagccaggcctgtccaaat  tcctactgt-ttcatgactaaccgcaattccctgatagctgtcgtctcctcagtccgtac  catcatgggctccatcttcgccgctgtcttcgtcgccatctacaccaataagctcccagg  ctacatgaccagcattgtagtaccagcgctgcaaaagacgtctctttctgcagaggctat  cagtgaagtactggcaacagcacctactgcgagccaggccgcactctccgctatcaaagg  tgtcactcctgagatcttgcgtgtcatcaacgccaaggtcgccgatgcttacggtcactc  ttatgcctttgtttactacaccgctgctgctctcggggctgtttgccttcttgcggcatt  gtgtctgcgtgacttcgatgtttacctgactgaccacgttgcccgcatg--  [4] 1L_Fp  atgacgaaacaagaagcatccccagatgacatctcgccaggtcacaataacccagacact  cccctctcaaacgaagatgaacttcgctccaagatcttaaccgctgagcgttccactctc  ccagaaggatatttcctcagccgggagttcgtcggaacgtttggcggtatcgcgttcagt  cttgcggcgacatactttgccttccaggctggtgccggtgccctaatcaacatcaaccag  cagatcggcccaagtgaaaactactacctgttctcaatcgtatggacggtctctcagccc  atctctatgctcttgtttggtcgtctctctgatatgtttggtcgccgaaaccttgctctt  ggtgccaacattcttggaataatcggcggtatcattgctgccacttcgcagagcatcaac  caactcattgcagcaaatgttcttttgggtcttgcctctggcattccgggttcttaccct  cttttgacgggcgagcttatgacaaataaactcaagtttcttggtactatcgttgttgtg  gtgcccaacatcatcgccactgggttcggtccgtatcttggccttcgacttagtattctt  agttcatggagatggatcttttacatctacatcatcttgatgggtatgcctccaagcctc  acgcaacgcaaggatgacaagaaccacgctaacaa---cttgtagtaatcggtactaccc  tctggtacttcttctaccaccctccttcgtttgtccaacttcacggaaccagcatcagcc  gcaaagccgagcttgcaaccatcgactggcttggcacttttcttcttgtctcgggtctcg  ttctcttccttcttgggatcgcctggggagggcaaccatataa---atggacatcaggtc  gcgtccttggtcttctgatccctggtgcaattttgtccatcgcattcgttctctatgagg  tatacggcaagcctgccaaaccaatcgtacccatgaagttcttcaaggatgtccgaggct  acacatgcgtcgtcatcatttccgcaatcactggctgccttcagacggccttgttcatct  tgtggccttctcaagtcgcatacatcttcggaagcaccaccaatggatgggaggagactg  cttggatgtcaagcgttgtgaactttgcagcttgggctggtatcatcgtcgtcgggcctc  tcttccacgtcatcaagcatttacgtctacagcttgttgtcggatctgcctggatgacag  ctttcctggcggccatgtcgaccatcacctactccaacaagggatcggctattgctttcg  cctttctcagcacctttcccatcgggtggggagaggtcatgactatgttgatggttcagt  acattgttccggaacgagatctaggtgttgcctttggtcagtagccaggcctgtccaaat  tcctactgt-ttcatgactaaccgcaattccctgatagctgtcgtctcctcagtccgtac  catcatgggctccatcttcgccgctgtcttcgtcgccatctacaccaataagctcccagg  ctacatgaccagcattgtagtaccagcgctgcaaaagacgtctctttctgcagaggctat  cagtgaagtactggcaacagcacctactgcgagccaggccgcactctccgctatcaaagg  tgtcactcctgagatcttgcgtgtcatcaacgccaaggtcgccgatgcttacggtcactc  ttatgcctttgtttactacaccgctgctgctctcggggctgtttgccttcttgcgacatt  gtgtctgcgtgacttcgatgtttacctgactgaccacgttgcccgcatg--  [5] 81L_Fp  atgacgaaacaagaagcatccccagatgacatctcgccaggtcacaataacccagacact  cccctctcaaacgaagatgaacttcgctccaagatcttaaccgctgagcgttccactctc  ccagaaggatatttcctcagccgggagttcgtcggaacgtttggcggtatcgcgttcagt  cttgcggcgacatactttgccttccaggctggtgccggtgccctaatcaacatcaaccag  cagatcggcccaagcgagaactactacctgttctcaatcgtatggacggtctctcagccc  atctctatgctcttgtttggtcgtctctctgatatgtttggtcgccgaaaccttgctctt  ggtgccaacattcttggaataatcggcggtatcattgctgccacttcgcagagcatcaac  caactcattgcagcaaatgttcttttgggtcttgcctctggcattccgggttcttaccct  cttttgacgggcgagcttatgacaaataaactcaagtttcttggtactatcgttgttgtg  gtgcccaacatcatcgccactgggttcggtccgtatcttggccttcgacttagtattctt  agttcatggagatggatcttttacatctacatcatcttgatgggtatgcctccaagcctc  acgcaacgcaaggatgacaagaaccacgctaacaa---cttgtagtaatcggtactaccc  tctggtacttcttctaccaccctccttcgtttgtccaacttcacggaaccagcatcagcc  gcaaagccgagcttgcaaccatcgactggcttggcacttttcttcttgtctcgggtctcg  ttctcttccttcttgggatcgcctggggagggcaaccatacaa---atggacatcaggtc  gcgtccttggtcttctgatccctggtgcaattttgtccatcgcattcgttctatatgagg  tatacggcaagcctgccaagccaatcgtacccatgaagttcttcaaggatgtccgaggct  acacatgcgtcgtcatcatttccgcaatcactggctgccttcagacagccttgttcatct  tgtggccttctcaagtcgcatacatctttggaagcaccaccaatggatgggaggagactg  cttggatgtcaagcgttgtgaactttgcagcttgggctggtatcatcatcgtcgggcctc  tcttccacgtcatcaagcatttacgtctacagcttgttgtcgggtctgcctggatgacag  ctttcctggcggccatgtcgaccatcacctactccaacaagggatcggctattgccttcg  cctttctcagcacctttcccatcgggtggggagaggtcatgactatgttgatggttcagt  acattgttccggaacgagatctcggtgttgcctttggtaagtagccaggcctgtccaaat  tcctactgt-ttcatgactgaccgcaattccctgatagctgtcgtctcctcagtccgtac  catcatgggctccatcttcgccgctgtcttcgtcgccatctacaccaacaagctcccagg  ctacatgaccagcattgtagtaccagcgctgcaaaagacgtctctttctgcagaggctat  cagtgaagtactggcaacagcacctactgcgagccaggccgcactctccgctataaaagg  tgtcactcctgagatcttgcgtgtcatcaacgccaaggtcgccgatgcttacggtcactc  atatgcctttgtttactacaccgctgctgctctcggggctgtttgccttcttgcggcatt  gtgtctgcgtgacttcgatgtttacctgactgaccacgttgcccccatg--  [6] 99L_Fp  atgacgaaacaagaagcatccccagatgacatctcgccaggtcacaataacccagacact  cccctctcaaacgaagatgaacttcgctccaagatcttaaccgctgagcgttccactctc  ccagaaggatatttcctcagccgggagttcgtcggaacgtttggcggtatcgcgttcagt  cttgcggcgacatactttgccttccaggctggtgccggtgccctaatcaacatcaaccag  cagatcggcccaagcgagaactactacctgttctcaatcgtatggacggtctctcagccc  atctctatgctcttgtttggtcgtctctctgatatgtttggtcgccgaaaccttgctctt  ggtgccaacattcttggaataatcggcggtatcattgctgccacttcgcagagcatcaac  caactcattgcagcaaatgttcttttgggtcttgcctctggcattccgggttcttaccct  cttttgacgggcgagcttatgacaaataaactcaagtttcttggtactatcgttgttgtg  gtgcccaacatcatcgccactgggttcggtccgtatcttggccttcgacttagtattctt  agttcatggagatggatcttttacatctacatcatcttgatgggtatgcctccaagcctc  acgcaacgcaaggatgacaagaaccacgctaacaa---cttgtagtaatcggtactaccc  tctggtacttcttctaccaccctccttcgtttgtccaacttcacggaaccagcatcagcc  gcaaagccgagcttgcaaccatcgactggcttggcacttttcttcttgtctcgggtctcg  ttctcttccttcttgggatcgcctggggagggcaaccatacaa---atggacatcaggtc  gcgtccttggtcttctgatccctggtgcaattttgtccatcgcattcgttctatatgagg  tatacggcaagcctgccaagccaatcgtacccatgaagttcttcaaggatgtccgaggct  acacatgcgtcgtcatcatttccgcaatcactggctgccttcagacagccttgttcatct  tgtggccttctcaagtcgcatacatctttggaagcaccaccaatggatgggaggagactg  cttggatgtcaagcgttgtgaactttgcagcttgggctggtatcatcatcgtcgggcctc  tcttccacgtcatcaagcatttacgtctacagcttgttgtcgggtctgcctggatgacag  ctttcctggcggccatgtcgaccatcacctactccaacaagggatcggctattgccttcg  cctttctcagcacctttcccatcgggtggggagaggtcatgactatgttgatggttcagt  acattgttccggaacgagatctcggtgttgcctttggtaagtagccaggcctgtccaaat  tcctactgt-ttcatgactgaccgcaattccctgatagctgtcgtctcctcagtccgtac  catcatgggctccatcttcgccgctgtcttcgtcgccatctacaccaacaagctcccagg  ctacatgaccagcattgtagtaccagcgctgcaaaagacgtctctttctgcagaggctat  cagtgaagtactggcaacagcacctactgcgagccaggccgcactctccgctataaaagg  tgtcactcctgagatcttgcgtgtcatcaacgccaaggtcgccgatgcttacggtcactc  atatgcctttgtttactacaccgctgctgctctcggggctgtttgccttcttgcggcatt  gtgtctgcgtgacttcgatgtttacctgactgaccacgttgcccgcatg--  [7] L7_Fp  atgacgaaacaagaagcatccccagatgacatctcgccaggtcacaataacccagacact  cccctctcaaacgaagatgaacttcgctccaagatcttaaccgctgagcgttccactctc  ccagaaggatatttcctcagccgggagttcgtcggaacgtttggcggtatcgcgttcagt  cttgcggcgacatactttgccttccaggctggtgccggtgccctaatcaacatcaaccag  cagatcggcccaagcgagaactactacctgttctcaatcgtatggacggtctctcagccc  atctctatgctcttgtttggtcgtctctctgatatgtttggtcgccgaaaccttgctctt  ggtgccaacattcttggaataatcggcggtatcattgctgccacttcgcagagcatcaac  caactcattgcagcaaatgttcttttgggtcttgcctctggcattccgggttcttaccct  cttttgacgggcgagcttatgacaaataaactcaagtttcttggtactatcgttgttgtg  gtgcccaacatcatcgccactgggttcggtccgtatcttggccttcgacttagtattctt  agttcatggagatggatcttttacatctacatcatcttgatgggtatgcctccaagcctc  acgcaacgcaaggatgacaagaaccacgctaacaa---cttgtagtaatcggtactaccc  tctggtacttcttctaccaccctccttcgtttgtccaacttcacggaaccagcatcagcc  gcaaagccgagcttgcaaccatcgactggcttggcacttttcttcttgtctcgggtctcg  ttctcttccttcttgggatcgcctggggagggcaaccatacaa---atggacatcaggtc  gcgtccttggtcttctgatccctggtgcaattttgtccatcgcattcgttctatatgagg  tatacggcaagcctgccaagccaatcgtacccatgaagttcttcaaggatgtccgaggct  acacatgcgtcgtcatcatttccgcaatcactggctgccttcagacagccttgttcatct  tgtggccttctcaagtcgcatacatctttggaagcaccaccaatggatgggaggagactg  cttggatgtcaagcgttgtgaactttgcagcttgggctggtatcatcatcgtcgggcctc  tcttccacgtcatcaagcatttacgtctacagcttgttgtcgggtctgcctggatgacag  ctttcctggcggccatgtcgaccatcacctactccaacaagggatcggctattgccttcg  cctttctcagcacctttcccatcgggtggggagaggtcatgactatgttgatggttcagt  acattgttccggaacgagatctcggtgttgcctttggtaagtagccaggcctgtccaaat  tcctactgt-ttcatgactgaccgcaattccctgatagctgtcgtctcctcagtccgtac  catcatgggctccatcttcgccgctgtcttcgtcgccatctacaccaacaagctcccagg  ctacatgaccagcattgtagtaccagcgctgcaaaagacgtctctttctgcagaggctat  cagtgaagtactggcaacagcacctactgcgagccaggccgcactctccgctataaaagg  tgtcactcctgagatcttgcgtgtcatcaacgccaaggtcgccgatgcttacggtcactc  atatgcctttgtttactacaccgctgctgctctcggggctgtttgccttcttgcggcatt  gtgtctgcgtgacttcgatgtttacctgactgaccacgttgcccgcatg--  [8] 58L_Fp  atgacgaaacaagaagcatccccagatgacatctcgccaggtcacaataacccagacact  cccctctcaaacgaagatgaacttcgctccaagatcttaaccgctgagcgttccactctc  ccagaaggatatttcctcagccgggagttcgtcggaacgtttggcggtatcgcgttcagt  cttgcggcgacatactttgccttccaggctggtgccggtgccctaatcaacatcaaccag  cagatcggcccaagcgagaactactacctgttctcaatcgtatggacggtctctcagccc  atctctatgctcttgtttggtcgtctctctgatatgtttggtcgccgaaaccttgctctt  ggtgccaacattcttggaataatcggcggtatcattgctgccacttcgcagagcatcaac  caactcattgcagcaaatgttcttttgggtcttgcctctggcattccgggttcttaccct  cttttgacgggcgagcttatgacaaataaactcaagtttcttggtactatcgttgttgtg  gtgcccaacatcatcgccactgggttcggtccgtatcttggccttcgacttagtattctt  agttcatggagatggatcttttacatctacatcatcttgatgggtatgcctccaagcctc  acgcaacgcaaggatgacaagaaccacgctaacaa---cttgtagtaatcggtactaccc  tctggtacttcttctaccaccctccttcgtttgtccaacttcacggaaccagcatcagcc  gcaaagccgagcttgcaaccatcgactggcttggcacttttcttcttgtctcgggtctcg  ttctcttccttcttgggatcgcctggggagggcaaccatacaa---atggacatcaggtc  gcgtccttggtcttctgatccctggtgcaattttgtccatcgcattcgttctatatgagg  tatacggcaagcctgccaagccaatcgtacccatgaagttcttcaaggatgtccgaggct  acacatgcgtcgtcatcatttccgcaatcactggctgccttcagacagccttgttcatct  tgtggccttctcaagtcgcatacatctttggaagcaccaccaatggatgggaggagactg  cttggatgtcaagcgttgtgaactttgcagcttgggctggtatcatcatcgtcgggcctc  tcttccacgtcatcaagcatttacgtctacagcttgttgtcgggtctgcctggatgacag  ctttcctggcggccatgtcgaccatcacctactccaacaagggatcggctattgccttcg  cctttctcagcacctttcccatcgggtggggagaggtcatgactatgttgatggttcagt  acattgttccggaacgagatctcggtgttgcctttggtaagtagccaggcctgtccaaat  tcctactgt-ttcatgactgaccgcaattccctgatagctgtcgtctcctcagtccgtac  catcatgggctccatcttcgccgctgtcttcgtcgccatctacaccaacaagctcccagg  ctacatgaccagcattgtagtaccagcgctgcaaaagacgtctctttctgcagaggctat  cagtgaagtactggcaacagcacctactgcgagccaggccgcactctccgctataaaagg  tgtcactcctgagatcttgcgtgtcatcaacgccaaggtcgccgatgcttacggtcactc  atatgcctttgtttactacaccgctgctgctctcggggctgtttgccttcttgcggcatt  gtgtctgcgtgacttcgatgtttacctgactgaccacgttgcccgcatg--  [9] 3L_Fp  atgacgaaacaagaagcatccccagatgacatctcgccaggtcacaataacccagacact  cccctctcaaacgaagatgaacttcgctccaagatcttaaccgctgagcgttccactctc  ccagaaggatatttcctcagccgggagttcgtcggaacgtttggcggtatcgcgttcagt  cttgcggcgacatactttgccttccaggctggtgccggtgccctaatcaacatcaaccag  cagatcggcccaagcgagaactactacctgttctcaatcgtatggacggtctctcagccc  atctctatgctcttgtttggtcgtctctctgatatgtttggtcgccgaaaccttgctctt  ggtgccaacattcttggaataatcggcggtatcattgctgccacttcgcagagcatcaac  caactcattgcagcaaatgttcttttgggtcttgcctctggcattccgggttcttaccct  cttttgacgggcgagcttatgacaaataaactcaagtttcttggtactatcgttgttgtg  gtgcccaacatcatcgccactgggttcggtccgtatcttggccttcgacttagtattctt  agttcatggagatggatcttttacatctacatcatcttgatgggtatgcctccaagcctc  acgcaacgcaaggatgacaagaaccacgctaacaa---cttgtagtaatcggtactaccc  tctggtacttcttctaccaccctccttcgtttgtccaacttcacggaaccagcatcagcc  gcaaagccgagcttgcaaccatcgactggcttggcacttttcttcttgtctcgggtctcg  ttctcttccttcttgggatcgcctggggagggcaaccatacaa---atggacatcaggtc  gcgtccttggtcttctgatccctggtgcaattttgtccatcgcattcgttctatatgagg  tatacggcaagcctgccaagccaatcgtacccatgaagttcttcaaggatgtccgaggct  acacatgcgtcgtcatcatttccgcaatcactggctgccttcagacagccttgttcatct  tgtggccttctcaagtcgcatacatctttggaagcaccaccaatggatgggaggagactg  cttggatgtcaagcgttgtgaactttgcagcttgggctggtatcatcatcgtcgggcctc  tcttccacgtcatcaagcatttacgtctacagcttgttgtcgggtctgcctggatgacag  ctttcctggcggccatgtcgaccatcacctactccaacaagggatcggctattgccttcg  cctttctcagcacctttcccatcgggtggggagaggtcatgactatgttgatggttcagt  acattgttccggaacgagatctcggtgttgcctttggtaagtagccaggcctgtccaaat  tcctactgt-ttcatgactgaccgcaattccctgatagctgtcgtctcctcagtccgtac  catcatgggctccatcttcgccgctgtcttcgtcgccatctacaccaacaagctcccagg  ctacatgaccagcattgtagtaccagcgctgcaaaagacgtctctttctgcagaggctat  cagtgaagtactggcaacagcacctactgcgagcaaggccgcactctccgctataaaagg  tgtcactcctgagatcttgcgtgtcatcaacgccaaggtcgccgatgcttacggtcactc  atatgcctttgtttactacaccgctgctgctctcggggctgtttgccttcttgcggcatt  gtgtctgcgtgacttcgatgtttacctgactgaccacgttgcccgcatg--  [10] 36L_Fp  atgacgaaacaagaagcatccccagatgacatctcgccaggtcacaataacccagacact  cccctctcaaacgaagatgaacttcgctccaagatcttgaccgctgagcgttccactctc  ccagaaggatatttcctcagccgggagttcgtcggaacgtttggcggtatcgcgttcagt  cttgcggcgacatactttgccttccaggctggtgccggtgccctaatcaacatcaaccag  cagatcggcccaagcgagaactactacctgttctcaatcgtatggacggtctctcagccc  atctctatgctcttgtttggtcgtctctctgatatgtttggtcgccgaaaccttgctctt  ggtgccaacattcttggaataatcggcggtatcattgctgccacttcgcagagcatcaac  caactcattgcagcaaatgttcttttgggtcttgcctctggcattccgggttcttaccct  cttttgacgggcgagcttatgacaaataaactcaagtttcttggtactatcgttgttgtg  gtgcccaacatcatcgccactgggttcggtccgtatcttggccttcgacttagtattctt  agttcatggagatggatcttttacatctacatcatcttgatgggtatgcctccaagcctc  acgcaacgcaaggatgacaagaaccacgctaacaa---cttgtagtaatcggtactaccc  tctggtacttcttctaccaccctccttcgtttgtccaacttcacggaaccagcatcagcc  gcaaagccgagcttgcaaccatcgactggcttggcacttttcttcttgtctcgggtctcg  ttctcttccttcttgggatcgcctggggagggcaaccatacaa---atggacatcaggtc  gcgtccttggtcttctgatccctggtgcaattttgtccatcgcattcgttctatatgagg  tatacggcaagcctgccaagccaatcgtacccatgaagttcttcaaggatgtccgaggct  acacatgcgtcgtcatcatttccgcaatcactggctgccttcagacagccttgttcatct  tgtggccttctcaagtcgcatacatctttggaagcaccaccaatggatgggaggagactg  cttggatgtcaagcgttgtgaactttgcagcttgggctggtatcatcatcgtcgggcctc  tcttccacgtcatcaagcatttacgtctacagcttgttgtcgggtctgcctggatgacag  ctttcctggcggccatgtcgaccatcacctactccaacaagggatcggctattgccttcg  cctttctcagcacctttcccatcgggtggggagaggtcatgactatgttgatggttcagt  acattgttccggaacgagatctcggtgttgcctttggtaagtagccaggcctgtccaaat  tcctactgt-ttcatgactgaccgcaattccctgatagctgtcgtctcctcagtccgtac  catcatgggctccatcttcgccgctgtcttcgtcgccatctacaccaacaagctcccagg  ctacatgaccagcattgtagtaccagcgctgcaaaagacgtctctttctgcagaggctat  cagtgaagtactggcaacagcacctactgcgagccaggccgcactctccgctataaaagg  tgtcactcctgagatcttgcgtgtcatcaacgccaaggtcgccgatgcttacggtcactc  atatgcctttgtttactacaccgctgctgctctcggggctgtttgccttcttgcggcatt  gtgtctgcgtgacttcgatgtttacctgactgaccacgttgcccgcatg--  [11] FFUJ_06844  atgacgaaacaagaagcctccccagatgacatctcgccaggtcacaatgaccagaacatt  gtcccctcaaacgaagatgaacttcgatccaagatcttaaccgctgagcgctcaaccctc  ccagaaggatacttcctcagccgagagttcgtcggaacgtttggcggcatcgcattcagt  cttgcagcgacatactttgccttccaggctggagccggcgccctcatcaacatcaaccag  cagatcggcccaagtgagaactactacctgttctcaatcgtatggacggtctctcagccc  atctctatgctcttgtttggtcgtctctctgatatgtttggtcgccgaaaccttgctctt  ggtgccaacattcttggaataatcggcggtatcattgctgccacttcgcagagcatcaac  caactcattgcagcaaatgttcttttgggtcttgcgtcgggtattccgggttcttaccct  cttttgaccggcgagcttatgacgaacaagctcaagtttcttggtactattgttgttgtg  gtgcccaacatcatcgccactgggttcggtccgtatcttggccttcgacttagtattctt  agttcatggagatggatcttttacatctacatcatcttgatgggtatgcctccaagcctc  acgcaaggcaaggatgacaagaaccacgctaacaa---cgtgtagtaatcggtactaccc  tctggtacttcttctaccaccctccttcgtttgtccaacttcacggaaccagcatcagcc  gcaaagccgagcttgcaaccatcgactggcttggcacttttcttcttgtctcgggtctcg  ttctcttccttcttgggatcgcctggggagggcaaccatacaa---atggacatcaggtc  gcgtccttggtcttctgatccctggtgcaattttgtccatagccttcattctctatgagg  tatacggcaagcctgccaagccaatcgtacccatgaagttcttcaaggatgtccgaggct  acacatgcgtcgtcatcatttccgcaatcactggctgccttcagacggccttgttcatct  tgtggccttctcaagtcgcatacatcttcggaagcaccaccaatggatgggaggagactg  cttggatgtcaagcgttgtgaactttgcagcttgggctggtatcatcatcgtcgggcctc  tcttccacgtcatcaagcatttacgtctacagcttgttgtcggatctgcttggatgacag  ctttcctggcggccatgtcgaccatcacctactccaacaagggatcggctattgctttcg  cctttctcagcacccttcccatcgggtggggagaggtcatgactatgttgatggttcagt  acattgttccggaacgagatctaggtgttgcctttggtaagtagccaggcctgtccaaat  tcttactgt-ttcatgactaaccgcaattccctgatagctgtcgtctcctcagtccgtac  catcatgggctccatcttcgccgctgtcttcgtcgccatctacaccaacaagctcccagg  ctacatgaccagcattgtaataccagcgctacaaaagacgtctctttctgcagaggctat  cagcgaagtactggcaacagcacctactgcgagccaggccgcactctccgctatcaaagg  tgtcactcctgagatcttgcgtgtcatcaacgccaaggtcgccgatgcttacggtcactc  atatgcctttgtttactacaccgctgctgctcttggggctgtatgccttcttgcggcatt  gtgtctgcgtgactttgatgtttacctgactgatcacgttgcccgcatg--  [12] 10L_Fo  atgacgaaacaagaagcatcacaggatgacatctctccaggtcacgatgaccaggacatt  gccctctcaaacgaagatgaacttcgatccaagatcttgaccgctgaacgctcaactcta  ccagacggctacttcctcagccgcgagttcgttgggacgtttggcggtatcgcgttcagt  cttgcagcgacatactttgccttccaggctggcgccggcgccctcattaacatcaaccag  cacatcggccccagtgagaactactacctattctcgattgtatggacggtttctcaacct  atctccatgctcttgtttggtcgtctctctgatatgtttggtcgccggaaccttgctctt  ggtgccaacatcctcggaatctgtggtggtattgttgctgccacttcccagagcatcaac  caactcattgctgccaatgtcctcttgggccttgcctcgggcatcccgggttcatatcct  cttttgacaggcgagcttatgacgaacaagctcaagtttcttggtactatcgttgttgta  gtgcccaacatcatcgccactggcttcggtccgtatattggccttcgactcagcattctt  agttcatggagatggatcttttacatctacatcatcttgatgggtatgcctgcaagccta  acataacgcaaggatgactagaaccattctgacattatctcacagtaatcggtactaccc  tctggtacttcttctatcatcctccttcttttgtgcaacttcatggaactagcatcagcc  gcagagccgagcttgcaactatcgactgggttggcacttttcttcttgtctcgggtctcg  ttcttttccttcttggaattgcctggggtgggcaaccatacaa---atggacctcaggcc  gggtccttggtctcctgattcctggtgcagtttgctctatcgcattcgttctctatgaga  tatatggcaagcctgccaagccaatcgtgcccatgaaattcttcaaggatgtccgaggct  acacatgcgtcgttatcatttctgccatcaccggctgccttcagacggccctgtttatct  tgtggccttcacaggttgcatacatctttggaagcaccactaatggatgggaggagaccg  cttggatgtcaagtgttgtaaactttgcagcttgggctggtatcatcatcgtcggacctc  tgtttcacgtcatcaagcatttgcgtctacagcttgttgtaggatctgcctggatgacag  ctttcctggcggccatgtctaccatcacttactccaacaagggttctgctattgcttttg  cctttctcagtacctttcccatcgggtggggagaagttatgactatgctgatggttcagt  acattgtcccggagcgagacttgggtgttgcctttggtaagtggccaaatctatcccgaa  acctcctttcttcatgactaacagtcttgttttcaaagctgtcgtctcatcagtccgtac  catcatgggctccatctttgccgctgtcttcgtcgccatctacaccaacaagctcccagg  ctacatggccagcattgtagtaccggcgctgcaaaagacatctctttctgcagacgctat  tagtgaagtactggcaacagcacccactgcgaatcaggcagcactctcagctatcaaagg  cgtcactcctgagatcttgcgtgtcatcaacgccaaagttgccgatgcttacggccactc  atacgcctttgtttactacactgctgctgctcttggggctgtatgcctctttgcggcaat  gtgtctacgtgactttgatgtttatctgacagaccacgttgcccgaatg--  [13] 11L_Fo  atgacgaaacaagaagcatcacaagatgacatctctccgggtcacaatgaccagggcatt  gcgctctcaaacgaggatgaacttcgctccaagatcttgaccgctgagcgctcaactctc  ccagacggctactttctcagccgcgagttcgttggaacgtttggcggcattgcgttcagt  cttgcagcgacatactttgccttccaggctggtgccggtgccctcataaacatcaaccag  cacatcggccccagtgagaactactacctattctcaattgtgtggacggtatctcaaccc  atctcgatgctcttgtttggccgtctctctgacatgtttggtcgccgaaaccttgctctt  ggtgccaatgtcctcggtatctgtggcggtattgttgctgccacttcccagagcatcaac  cagctcattgctgccaatgttctcttgggtcttgcctcgggcattccaggttcatatcct  cttttgacaggcgagcttatgacgaacaagctcaagtttcttggtactatcgttgttgta  gtgcccaatatcatcgccactggcttcggtccgtacctaggccttcgacttagcattctt  agttcatggagatggatcttttacatctacatcatcttaatgcgtatgcctgcaaacttg  acacaacgcaaggataactagaaccatgctgacaatatctcacagtaatcggtactaccc  tctggtacttcttctaccaccctccttcttttgtgcaacttcacggaacaagcatcagcc  gaaaggccgagcttgcaactatcgactgggttggcacttttcttcttgtctcgggccttg  ttctcttccttcttggaattgcctggggtgggcaaccgtacaa---atggacctcaggtc  gggtccttggtctcctgattcctggtgcagtctgctctatcgcattcgttctttatgaga  tatatggccagcctgcgaagcctatcgtacccatgaaattctttaaggatgtccgaggtt  acacatgcgtcgtcattatttccgccatcactggctgccttcaaacagcgctattcatat  tgtggccttcacaggttgcatacatctttggaagcaccaccaacggatgggaggagaccg  cttggatgtcaagtgttgtaaactttgcagcttgggctggtatcatcatcgtcgggcctc  tgtttcacgtcatcaagcatttgcgtctacagcttgttgtcggatctgcctggatgacag  ctttcctggcggccatgtctaccatcacttactccaacaagagttctgctattgcttttg  cctttctcagcacctttcccatcgggtggggagaagtcatgactatgctgatggttcagt  acattgttccggaacgagatctgggggttgcctttggtaagtaaccaaatctatcccgaa  ccctcctttcttcatgactaacagtcttctttccaaagctgtcgtctcatcagttcgtac  catcatgggctccatcttcgccgctgtcttcgtcgccatctacaccaacaagcttccggg  ctacatggccagcattgtagtaccggcgctgcaaaagacatctctctctgcagatgctat  tagtgaagtactagcgacagcacccactgcgaatcaggcagcactctcagctatcaaagg  tgtcactcctgagatcttgcgtgtcatcaacgccaaagtcgccgatgcttacggccactc  gtacgcctttgtttactacaccgctgctggtctcggggttgtatgcttctttgcggcaat  gtgtctgcgcgactttgatgtttatctgacagaccacgttgcccgaatg--  [14] 131L_Fo  atgacgaaacaagaagcatcacaagatgacatctctccgggtcacaatgaccaggacatt  gcgctctcaaacgaggatgaacttcgttccaagatcttgaccgctgagcgctcaactctc  ccagatggctacttcctcagccgcgagttcgttggaacgttcggcggcattgcgttcagt  cttgcagcgacatactttgccttccaggctggtgccggtgccctcatcaacatcaaccag  cacatcggccccagtgagaactactacctattctcaattgtgtggacggtctctcaaccc  atctcgatgctcttgtttggccgtctctctgacatgtttggtcgccgaaaccttgctctt  ggtgccaatgtcctcggtatctgtggtggtattgttgctgccacttcccagagcatcaac  cagctcattgctgccaatgttctcttgggtcttgcctcgggcattccgggttcatatcct  cttttgacaggcgagcttatgacgaacaagctcaagtttcttggtactatcgttgttgta  gtgcccaatatcatcgccactggcttcggtccgtacctaggccttcgacttagcattctt  agttcatggagatggatcttttacatctacatcatcttaatgggtatgcctgcagacttg  acacaacgcaaggataactagaaccatgctgacaatatctcacagtaatcggtactaccc  tctggtacttcttctaccatcctccttcttttgtgcaacttcacggaacaagcatcagcc  gaaaggccgagcttgcaactatcgactgggttggcacttttcttcttgtctcgggccttg  ttctcttccttcttggaattgcctggggtgggcaaccgtacaa---atggacctcaggtc  gggtccttggtctcctgattcctggtgcagtttgctctatcgcattcgttctttatgaga  tatatggccagcctgcgaagcctatcgtacccatgaaattctttaaggatgtccgaggtt  acacatgcgtcgtcatcatttccgccatcactggctgccttcaaacagcgctcttcatat  tgtggccttcacaggttgcatacatctttggaagcaccaccaacggatgggaggagaccg  cttggatgtcaagtgttgtaaactttgcagcttgggccggtatcatcatcgtcgggcctc  tgtttcacgtcatcaagcatttgcgtctacagcttgttgtcggatctgcctggatgacag  ctttcctggcggccatgtctaccatcacttactccaacaagggttctgctattgcttttg  cctttctcagcacctttcccatcgggtggggagaagtcatgactatgctgatggttcagt  acattgttccggaacgagatctgggggttgcctttggtaagtaaccaaatctatcccgaa  ccctcctttcttcatgactaacagtcttctttccaaagctgtcgtctcatcagtccgtac  catcatgggctccatcttcgccgctgtcttcgtcgccatctacaccaacaagcttccggg  ctacatggccagcattgtagtaccggcgctgcaaaagacatctctttctgcagatgctat  taatgaagtactagcgacagcacccactgcgaatcaggcagcactctcagctatcaaagg  cgtcactcctgagatcttgcgtgtcatcaacgccaaagtcgccgatgcttacggccactc  gtacgcctttgtttactacaccgctgctgctcttggggctgtatgcttctttgcggcaat  gtgtctgcgcgactttgatgtttatctgacagaccacgttgcccgaatg--  [15] 55L_Fo  atgacgaaacaagaagtatcacaagatgacatctctccgggtcacaatgaccaggacatt  gcgctctcaaacgaggatgaacttcgttccaagatcttgaccgctgagcgctcaactctc  ccagatggctacttcctcagccgcgagttcgttggaacgttcggcggcattgcgttcagt  cttgcagcgacatactttgccttccaggctggtgccggtgccctcataaacatcaaccag  cacatcggccccagtgagaactactacctattttcaattgtgtggacggtctctcaaccc  atctcgatgctcttgtttggccgtctctctgacatgtttggtcgccgaaaccttgctctt  ggtgccaatgtcctcggtatctgtggtggtattgttgctgccacttcccagagcatcaac  cagctcattgctgccaatgttctcttgggtcttgcctcgggcattccgggttcatatcct  cttttgacaggcgagcttatgacgaacaagctcaagtttcttggtactatcgttgttgta  gtgcccaatatcatcgccactggcttcggtccgtacctaggccttcgacttagcattctt  agttcatggagatggatcttttacatctacatcatcttaatgggtatgcctgcaaacttg  acacaacgcaaggataactaaaaccatgctgacaatatctcacagtaatcggtactaccc  tctggtacttcttctaccatcctccttcttttgtgcaacttcacggaacaagcatcagcc  gaaaggccgagcttgcaactatcgactgggttggcacttttcttcttgtctcgggccttg  ttctcttccttcttggaattgcctggggtgggcaaccgtacaa---atggacctcaggtc  gggtccttggtctcctgattcctggtgcagtttgctctatcgcattcgttctttatgaga  tatatggccagcctgcgaagcccattgtacccatgaaattctttaaggatgtccgaggtt  acacatgcgtcgtcatcatttccgccatcactggctgccttcaaacagcgctattcatat  tgtggccttcacaggttgcatacatctttggaagcaccaccaacggatgggaggagaccg  cttggatgtcaagtgttgtaaactttgcagcttgggctggtatcatcatcgtcgggcctc  tgtttcacgtcatcaagcatttgcgtctacagcttgttgtcggatctgcctggatgacag  ctttcctggcggccatgtctaccatcacttactccaacaagggttctgctattgcttttg  cctttctcagcacctttcccatcgggtggggagaagtcatgactatgctgatggttcagt  acattgttccggaacgagatctgggggttgcctttggtaagtgaccaaatctatcccgaa  ccctcctttcttcatgactaacagtcttctttccaaagctgtcgtctcatcagtccgtac  catcatgggctccattttcgccgctgtcttcgtcgccgtctacaccaacaagcttccggg  ctacatggccagcattgtagtaccggcgctgcaaaatacatctctttctgcagatgctat  tagtgaagtactagcgacagcacccactgcgaatcaggcagcactctcagctatcaaagg  cgttactcctgagatcttgcgtgtcatcaacgccaaagtcgccgatgcttacggccactc  gtacgcctttgtttactacaccgctgctgctctcggggctgtatgcttctttgcggcaat  gtgtctgcgcgactttgatgtttatctgacagaccacgttgcccgaatg--  [16] 57L_Fo  atgacgaaacaagaagcatcacaagatgacatctctccgggtcacaatgaccaggacatt  gcgctctcaaacgaggatgaacttcgttccaagatcttgaccgctgagcgctcaactctc  ccagacggctacttcctcagccgcgagttcgttggaacattcggcggcattgctttcagt  cttgcagcgacatattttgccttccaggctggtgccggtgcactcatcaacatcaaccag  cacatcggccccagtgagaattactacctattctcaattgtgtggacggtctctcaaccc  atctcgatgctcttgtttggccgtctctctgacatgtttggtcgccgaaaccttgctctt  ggtgccaatgtcctcggtatctgtggtggtattgttgctgccacttcccagagcatcaac  cagctcattgctgccaatgttctcttgggtcttgcctcgggcattccgggttcatatcct  cttttgacaggcgagcttatgacgaacaaactcaagtttcttggtactatcgttgttgta  gtgcccaatatcatcgccactggcttcggtccgtacctaggccttcgacttagcattctt  agttcatggagatggatcttttacatctacatcatcttaatgggtatgcctgcaaacttg  acacaacgcatggataactagaaccatgctgacaatatctcacagtaatcggtactaccc  tctggtacttcttctaccatcctccttcttttgtgcaacttcacggaacaagtatcagcc  gaaaggccgagcttgcaactatcgactgggttggcacttttcttcttgtctcgggccttg  ttctcttccttcttggaattgcctggggtgggcaaccgtacaa---atggacctcaggtc  gggtccttggtctcctgattcctggtgcagtttgctcgatcgcattcgttctttatgaga  tatatggccagcctgcgaagcctatcgtacccatgaaattctttaaggatgtccgaggtt  acacatgcgtcgtcatcatttccgccatcactggctgccttcaaacagcgctattcatat  tgtggccttcacaggttgcatacatctttggaagcaccaccaacggatgggaggagaccg  cttggatgtcaagtgttgtaaactttgcagcttgggccggtatcatcatcgtcgggcctc  tgtttcacgtcatcaagcatttgcgtctacagcttgttgtcggatctgcctggatgacag  ctttcctggcggccatgtctaccatcacttactccaacaagggttctgctattgcttttg  cctttctcagcacctttcccatcgggtggggagaagtcatgactatgctgatggttcagt  acattgttccggaacgagatctgggggttgcctttggtaagtaaccaaatctatcccgaa  ccctcctttcttcatgactaacagtcttctttccaaagctgtcgtctcatcagtccgtac  catcatgggctccatcttcgccgctgtcttcgtcgccatctacaccaacaagcttccggg  ctacatggccagcattgtagtaccggcgctgcaaaagacatctctttctacagatgctat  tagtgaagtactagcgacagcacccactgcgaatcaggcagcactctcagctatcaaagg  cgtcactcctgagatcttgcgtgtcatcaacgccaaagtcgccgatgcttacggccactc  gtacgcctttgtttactacaccgctgctgctcttggggctgtatgcttctttgcggcaat  gtgtctgcgcgactttgatgtttatctgacagaccacgttgcccgaatg--  [17] 19L_Fo  atgacgaaacaagaagcatcacaagatgacatctctccgggtcacaatgaccaggacatt  gcgctctcaaacgaggatgaacttcgttccaagatcttgaccgctgagcgctcaactctc  ccagacggctacttcctcagccgcgagttcgttggaacattcggcggcattgctttcagt  cttgcagcgacatattttgccttccaggctggtgccggtgcactcatcaacatcaaccag  cacatcggccccagtgagaattactacctattctcaattgtgtggacggtctctcaaccc  atctcgatgctcttgtttggccgtctctctgacatgtttggtcgccgaaaccttgctctt  ggtgccaatgtcctcggtatctgtggtggtattgttgctgccacttcccagagcatcaac  cagctcattgctgccaatgttctcttgggtcttgcctcgggcattccgggttcatatcct  cttttgacaggcgagcttatgacgaacaaactcaagtttcttggtactatcgttgttgta  gtgcccaatatcatcgccactggcttcggtccgtacctaggccttcgacttagcattctt  agttcatggagatggatcttttacatctacatcatcttaatgggtatgcctgcaaacttg  acacaacgcatggataactagaaccatgctgacaatatctcacagtaatcggtactaccc  tctggtacttcttctaccatcctccttcttttgtgcaacttcacggaacaagtatcagcc  gaaaggccgagcttgcaactatcgactgggttggcacttttcttcttgtctcgggccttg  ttctcttccttcttggaattgcctggggtgggcaaccgtacaa---atggacctcaggtc  gggtccttggtctcctgattcctggtgcagtttgctcgatcgcattcgttctttatgaga  tatatggccagcctgcgaagcctatcgtacccatgaaattctttaaggatgtccgaggtt  acacatgcgtcgtcatcatttccgccatcactggctgccttcaaacagcgctattcatat  tgtggccttcacaggttgcatacatctttggaagcaccaccaacggatgggaggagaccg  cttggatgtcaagtgttgtaaactttgcagcttgggccggtatcatcatcgtcgggcctc  tgtttcacgtcatcaagcatttgcgtctacagcttgttgtcggatctgcctggatgacag  ctttcctggcggccatgtctaccatcacttactccaacaagggttctgctattgcttttg  cctttctcagcacctttcccatcgggtggggagaagtcatgactatgctgatggttcagt  acattgttccggaacgagatctgggggttgcctttggtaagtaaccaaatctatcccgaa  ccctcctttcttcatgactaacagtcttctttccaaagctgtcgtctcatcagtccgtac  catcatgggctccatcttcgccgctgtcttcgtcgccatctacaccaacaagcttccggg  ctacatggccagcattgtagtaccggcgctgcaaaagacatctctttctacagatgctat  tagtgaagtactagcgacagcacccactgcgaatcaggcagcactctcagctatcaaagg  cgtcactcctgagatcttgcgtgtcatcaacgccaaagtcgccgatgcttacggccactc  gtacgcctttgtttactacaccgctgctgctcttggggctgtatgcttctttgcggcaat  gtgtctgcgcgactttgatgtttatctgacagaccacgttgcccgaatg--  [18] 94L_Fo  atgacgaaacaagaagcatcacaagatgacatctctccgggtcacaatgaccagggcatt  gcgctctcaaacgaggatgaacttcgctccaagatcttgaccgctgagcgctcaactctc  ccagacggctactttctcagccgcgagttcgttggaacgtttggcggcattgcgttcagt  cttgcagcgacatactttgccttccaggctggtgccggtgccctcataaacatcaaccag  cacatcggccccagtgagaactactacctattctcaattgtgtggacggtatctcaaccc  atctcgatgctcttgtttggccgtctctctgacatgtttggtcgccgaaaccttgctctt  ggtgccaatgtcctcggtatctgtggcggtattgttgctgccacttcccagagcatcaac  cagctcattgctgccaatgttctcttgggtcttgcctcgggcattccaggttcatatcct  cttttgacaggcgagcttatgacgaacaagctcaagtttcttggtactatcgttgttgta  gtgcccaatatcatcgccactggcttcggtccgtacctaggccttcgacttagcattctt  agttcatggagatggatcttttacatctacatcatcttaatgcgtatgcctgcaaacttg  acacaacgcaaggataactagaaccatgctgacaatatctcacagtaatcggtactaccc  tctggtacttcttctaccaccctccttcttttgtgcaacttcacggaacaagcatcagcc  gaaaggccgagcttgcaactatcgactgggttggcacttttcttcttgtctcgggccttg  ttctcttccttcttggaattgcctggggtgggcaaccgtacaa---atggacctcaggtc  gggtccttggtctcctgattcctggtgcagtctgctctatcgcattcgttctttatgaga  tatatggccagcctgcgaagcctatcgtacccatgaaattctttaaggatgtccgaggtt  acacatgcgtcgtcattatttccgccatcactggctgccttcaaacagcgctattcatat  tgtggccttcacaggttgcatacatctttggaagcaccaccaacggatgggaggagaccg  cttggatgtcaagtgttgtaaactttgcagcttgggctggtatcatcatcgtcgggcctc  tgtttcacgtcatcaagcatttgcgtctacagcttgttgtcggatctgcctggatgacag  ctttcctggcggccatgtctaccatcacttactccaacaagagttctgctattgcttttg  cctttctcagcacctttcccatcgggtggggagaagtcatgactatgctgatggttcagt  acattgttccggaacgagatctgggggttgcctttggtaagtaaccaaatctatcccgaa  ccctcctttcttcatgactaacagtcttctttccaaagctgtcgtctcatcagttcgtac  catcatgggctccatcttcgccgctgtcttcgtcgccatctacaccaacaagcttccggg  ctacatggccagcattgtagtaccggcgctgcaaaagacatctctctctgcagatgctat  tagtgaagtactagcgacagcacccactgcgaatcaggcagcactctcagctatcaaagg  tgtcactcctgagatcttgcgtgtcatcaacgccaaagtcgccgatgcttacggccactc  gtacgcctttgtttactacaccgctgctggtctcggggttgtatgcttctttgcggcaat  gtgtctgcgcgactttgatgtttatctgacagaccacgttgcccgaatg--  [19] 115L_Fo  atgacgaaacaagaagcatcacaagatgacatctctccgggtcacaatgaccaggacgtt  gcactctcaaacgaagatgaacttcgttccaagatcttgactgctgagcgctcaactctc  ccagatggctacttcctcagccgcgagttcgttggaacattcggcggcattgctttcagt  cttgcagcgacatattttgccttccaggctggtgccggtgcactcatcaacatcaaccag  cacatcggccccagtgagaattactacctattctcaattgtgtggacggtctctcaaccc  atctcgatgctcttgtttggccgtctctctgacatgtttggtcgccgaaaccttgctctt  ggtgccaatgtcctcggtatctgtggtggtattgttgctgccacttcccagagcatcaac  cagctcattgctgccaatgttctcttgggtcttgcctcgggcattccgggttcatatcct  cttttgacaggcgagcttatgacgaacaaactcaagtttcttggtactatcgttgttgta  gtgcccaatatcatcgccactggcttcggtccgtacctaggccttcgacttagcattctt  agttcatggagatggatcttttacatctacatcatcttaatgggtatgcctgcaaacttg  acacaacgcaaggataactagaaccatgctgacaatatctcacagtaatcggtactaccc  tctggtacttcttctaccatcctccttcttttgtgcaacttcacggaacaagcatcagcc  gaaaggccgagcttgcaactatcgactgggttggcacttttcttcttgtctcgggccttg  ttctcttccttcttggaattgcctggggtgggcaaccgtacaa---atggacttcaggtc  gggtccttggtctcctgattcctggtgcagtttgctctatcgcattcgttctttatgaga  tatatggccagcctgcgaagcctatcgtacccatgaaattctttaaggatgtccgaggtt  acacatgcgtcgtcatcatttccgccatcactggctgccttcaaacagcgctattcatat  tgtggccttcacaggttgcatacatctttggaagcaccaccaacggatgggaggagaccg  cttggatgtcaagtgttgtaaactttgcagcttgggccggtatcatcatcgtcgggcctc  tgtttcacgtcatcaagcatttgcgtctacagcttgttgtcggatctgcctggatgacag  ctttcctggcggccatgtctaccatcacttactccagcaagggttctgctattgcttttg  cctttctcagcacctttcccatcgggtggggagaagtcatgactatgctgatggttcagt  acattgttccggaacgagatctgggggttgcctttggtaagtaaccaaatctatcccgaa  ccctcctttcttcatgactaacagtcttctttccaaagctgtcgtctcatcagtccgtac  catcatgggctccatcttcgccgctgtcttcgtcgccatctacaccaacaagcttccggg  ctacatggccagcattgtagtaccggcgctgcaaaagacatctctttctgcagatgctat  tagtgaagtactagcgacagcacccactgcgaatcaggcagcactctcagctatcaaagg  cgtcactcctgagatcttgcgtgtcatcaacgccaaagtcgccgatgcttacggccactc  gtacgcctttgtttactacaccgctgctgctcttggggctgtatgcttctttgcggcaat  gtgtctgcgcgactttgatgtttatctgacagaccacgttgcccgaatg--  [20] FOPG_13450__Fusarium_oxysporum_f._sp._conglutinans_race_2_54008  atgacgaaacaagaagcatcacaagatgacatctctccgggtcacaatgaccaggacgtt  gcactctcaaacgaagatgaacttcgttccaagatcttgactgctgagcgctcaactctc  ccagatggctacttcctcagccgcgagttcgttggaacatttggcggcattgctttcagt  cttgcagcgacatattttgccttccaggctggtgccggtgcactcatcaacatcaaccag  cacatcggccccagtgagaattactacctattctcaattgtgtggacggtctctcaaccc  atctcgatgctcttgtttggccgtctctctgacatgtttggtcgccgaaaccttgctctt  ggtgccaatgtcctcggtatctgtggtggtattgttgctgccacttcccagagcatcaac  cagctcattgctgccaatgttctcttgggtcttgcctcgggcattccgggttcatatcct  cttttgacaggcgagcttatgacgaacaaactcaagtttcttggtactatcgttgttgta  gtgcccaatatcatcgccactggcttcggtccgtacctaggccttcgacttagcattctt  agttcatggagatggatcttttacatctacatcatcttaatgggtatgcctgcaaacttg  acacaacgcaaggataactagaaccatgctgacaatatctcacagtaatcggtactaccc  tctggtacttcttctaccatcctccttcttttgtgcaacttcacggaacaagcatcagcc  gaaaggccgagcttgcaactatcgactgggttggcacttttcttcttgtctcgggccttg  ttcccttccttcttggaattgcctggggtgggcaaccgtacaa---atggacctcaggtc  gggtccttggtctcctgattcctggtgcagtttgctctatcgcattcgttctttatgaga  tatatggccagcctgcgaagcctatcgtacccatgaaattctttaaggatgtccgaggtt  acacatgcgtcgtcatcatttccgccatcactggctgccttcaaacagcgctattcatat  tgtggccttcacaggttgcatacatctttggaagcaccaccaacggatgggaggagaccg  cttggatgtcaagtgttgtaaactttgcagcttgggctggtatcatcatcgtcgggcctc  tgtttcacgtcatcaagcatttgcgtctacagcttgttgtcggatctgcctggatgacag  ctttcctggcggccatgtctaccatcacttactccaacaagggttctgctattgcttttg  cctttctcagcacctttcccatcgggtggggagaagtcatgactatgctgatggttcagt  acattgttccggaacgagatctgggggttgcctttggtaagtaaccaaatctatcccgaa  ccctcctttcttcatgactaacagtcttctttccaaagctgtcgtctcatcagtccgtac  catcatgggctccatcttcgccgctgtcttcgtcgccatctacaccaacaagcttccggg  ctacatggccagcattgtagtaccggcgctgcaaaagacatctctttctgcagatgctat  tagtgaagtactagcgacagcacccactgcgaatcaggcagcactctcagctatcaaagg  cgtcactcctgagatcttgcgtgtcatcaacgccaaagtcgccgatgcttacggccactc  gtatgcctttgtttactacaccgctgctgctctcggggctgtatgcttctttgcggcaat  gtgtctgcgcgactttgatgtttatctgacagaccacgttgcccgaatg--  [21] FOC4_g10013206__Fusarium_oxysporum_f._sp._cubense_race_4  atgacgaaacaagaagcatcacaagatgacatctctccgggtcacaatgaccagggcatt  gcgctctcaaacgaggatgaacttcgctccaagatcttgaccgctgagcgctcaactctc  ccagacggctactttctcagccgcgagttcgttggaacgtttggcggcattgcgttcagt  cttgcagcgacatactttgccttccaggctggtgccggtgccctcataaacatcaaccag  cacatcggccccagtgagaactactacctattctcaattgtgtggacggtatctcaaccc  atctcgatgctcttgtttggccgtctctctgacatgtttggtcgccgaaaccttgctctt  ggtgccaatgtcctcggtatctgtggcggtattgttgctgccacttcccagagcatcaac  cagctcattgctgccaatgttctcttgggtcttgcctcgggcattccaggttcatatcct  cttttgacaggcgagcttatgacgaacaagctcaagtttcttggtactatcgttgttgta  gtgcccaatatcatcgccactggcttcggtccgtacctaggtcttcgacttagcattctt  agttcatggagatggatcttttacatctacatcatcttaatgcgtatgcctgcaaacttg  acacaacgcaaggataattagaaccatgctgacaatatctcacagtaatcggtactaccc  tctggtacttcttctaccaccctccttcttttgtgcaacttcacggaacaagcatcagcc  gaaaggccgagcttgcaactatcgactgggttggcacttttattcttgtctcgggccttg  ttctcttccttcttggaattgcctggggta------------------------------  -------------------------------------------------ttgatgtagga  tatttgtcctgc------------------------------------------------  ------------------------------------------------------------  -----------------------------------------------------gggaatg  cttaa-------------------------------------------------------  ------------------------------------------------------------  ------------------------------------------------------------  ------------------------------------------------------------  ------------------------------------------------------------  ------------------------------------------------------------  ------------------------------------------------------------  ------------------------------------------------------------  ------------------------------------------------------------  ------------------------------------------------------------  ------------------------------------------------------------  ---------------------------------------------------  [22] FOIG_09500__Fusarium_oxysporum_f._sp._cubense_tropical_race_4_54006  atgacgaaacaagaagcatcacaagatgacatctctccgggtcacaatgaccagggcatt  gcgctctcaaacgaggatgaacttcgctccaagatcttgaccgctgagcgctcaactctc  ccagacggctactttctcagccgcgagttcgttggaacgtttggcggcattgcgttcagt  cttgcagcgacatactttgccttccaggctggtgccggtgccctcataaacatcaaccag  cacatcggccccagtgagaactactacctattctcaattgtgtggacggtatctcaaccc  atctcgatgctcttgtttggccgtctctctgacatgtttggtcgccgaaaccttgctctt  ggtgccaatgtcctcggtatctgtggcggtattgttgctgccacttcccagagcatcaac  cagctcattgctgccaatgttctcttgggtcttgcctcgggcattccaggttcatatcct  cttttgacaggcgagcttatgacgaacaagctcaagtttcttggtactatcgttgttgta  gtgcccaatatcatcgccactggcttcggtccgtacctaggtcttcgacttagcattctt  agttcatggagatggatcttttacatctacatcatcttaatgcgtatgcctgcaaacttg  acacaacgcaaggataattagaaccatgctgacaatatctcacagtaatcggtactaccc  tctggtacttcttctaccaccctccttcttttgtgcaacttcacggaacaagcatcagcc  gaaaggccgagcttgcaactatcgactgggttggcacttttattcttgtctcgggccttg  ttctcttccttcttggaattgcctggggta------------------------------  -------------------------------------------------ttgatgtagga  tatttgtcctgc------------------------------------------------  ------------------------------------------------------------  -----------------------------------------------------gggaatg  cttaa-------------------------------------------------------  ------------------------------------------------------------  ------------------------------------------------------------  ------------------------------------------------------------  ------------------------------------------------------------  ------------------------------------------------------------  ------------------------------------------------------------  ------------------------------------------------------------  ------------------------------------------------------------  ------------------------------------------------------------  ------------------------------------------------------------  ---------------------------------------------------  [23] FOVG_05602__Fusarium_oxysporum_f._sp._pisi_HDV247  ------------------------------------------------------------  ------------------------------------------------------------  ------------------------------------------------------------  ------------------------------------------------------------  ------------------------------------------------------------  ------atgctcttgtttggccgtctctctgacatgtttggtcgccgaaaccttgctctt  ggtgccaatgtcctcggtatctgtggtggtattgttgctgccacttcccagagcatcaac  cagctcattgctgccaatgttctcttgggtcttgcctcgggcattccgggttcatatcct  cttttgacaggcgagcttatgacgaacaagctcaagtttcttggtactatcgttgttgta  gtgcccaatatcatcgccactggcttcggtccgtatctaggccttcgacttagcattctt  agttcatggagatggatcttttacatctacatcatcttaatgggtatgcctgcaaacttg  acacaacgcaaggataactagaaccatgctgacaatatctcacagtaatcggtactaccc  tctggtacttcttctaccatcctccttcttttgtgcaactccacggaacaagcatcagcc  gaaaggccgagcttgcaactatcgactgggttggcacttttcttcttgtctcgggccttg  ttctcttccttcttggaattgcctggggtgggcaaccgtacaa---atggacctcaggtc  gggttcttggtctcctgattcctggtgcagtttgctctatcgcattcgttctttatgaga  tatatggccagcctgcaaagcctatcgtacccatgaaattctttaaggatgtccgaggtt  acacatgcgtcgtcatcatttccgccatcactggctgccttcaaacagcgctattcatat  tatggccttcacaggttgcatacatctttggaagcaccaccaacggatgggaggagaccg  cttggatgtcaagtgttgtaaactttgcagcttgggctggtatcatcatcgtcgggcctc  tgtttcacgtcatcaagcatttgcgtctacagcttgttgtcggatctgcctggatgacag  ctttcctggcggccatgtctaccatcacttactctaacaagggttctgctattgcttttg  cctttctcagcacctttcccatcgggtggggagaagtcatgactatgctgatggttcagt  acattgttccggaacgagatctgggggttgcctttggtaagtaaccaaatctatcccgaa  ccctcctttcttcatgactaacagtcttctttccaaagctgtcgtctcatcagtccgtac  catcatgggctccatcttcgccgctgtcttcgtcgccatctacaccaacaagcttccggg  ctacatggccagcattgtagtaccggcgctgcaaaagacatctctttctgcagatgctat  tagtgaagtactagcgacagcacccactgcgaatcaggcagcactctcagctatcaaagg  cgtcactcctgagatcttgcgtgtcatcaacgccaaagtcgccgatgcttacggccactc  gtacgcctttgtttactacaccgctgctgctcttggggctgtatgcttctttgcggcaat  gtgtctgcgcgactttgatgtttatctgacagaccacgttgcccgaatg--  [24] FOQG_14213__Fusarium_oxysporum_f._sp._raphani_54005  atgacgaaacaagaagcatcacaagatgacatctctccgggtcacaatgaccaggacatt  gcgctctcaaacgaggatgaacttcgttccaagatcttgaccgctgagcgctcaactctc  ccagacggctacttcctcagccgcgagttcgttggaacattcggcggcattgctttcagt  cttgcagcgacatattttgccttccaggctggtgccggtgcactcatcaacatcaaccag  cacatcggccccagtgagaattactacctattctcaattgtgtggacggtctctcaaccc  atctcgatgctcttgtttggccgtctctctgacatgtttggtcgccgaaaccttgctctt  ggtgccaatgtcctcggtatctgtggtggtattgttgctgccacttcccagagcatcaac  cagctcattgctgccaatgttctcttgggtcttgcctcgggcattccgggttcatatcct  cttttgacaggcgagcttatgacgaacaaactcaagtttcttggtactatcgttgttgta  gtgcccaatatcatcgccactggcttcggtccgtacctaggccttcgacttagcattctt  agttcatggagatggatcttttacatctacatcatcttaatgggtatgcctgcaaacttg  acacaacgcatggataactagaaccatgctgacaatatctcacagtaatcggtactaccc  tctggtacttcttctaccatcctccttcttttgtgcaacttcacggaacaagtatcagcc  gaaaggccgagcttgcaactatcgactgggttggcacttttcttcttgtctcgggccttg  ttctcttccttcttggaattgcctggggtgggcaaccgtacaa---atggacctcaggtc  gggtccttggtctcctgattcctggtgcagtttgctcgatcgcattcgttctttatgaga  tatatggccagcctgcgaagcctatcgtacccatgaaattctttaaggatgtccgaggtt  acacatgcgtcgtcatcatttccgccatcactggctgccttcaaacagcgctattcatat  tgtggccttcacaggttgcatacatctttggaagcaccaccaacggatgggaggagaccg  cttggatgtcaagtgttgtaaactttgcagcttgggccggtatcatcatcgtcgggcctc  tgtttcacgtcatcaagcatttgcgtctacagcttgttgtcggatctgcctggatgacag  ctttcctggcggccatgtctaccatcacttactccaacaagggttctgctattgcttttg  cctttctcagcacctttcccatcgggtggggagaagtcatgactatgctgatggttcagt  acattgttccggaacgagatctgggggttgcctttggtaagtaaccaaatctatcccgaa  ccctcctttcttcatgactaacagtcttctttccaaagctgtcgtctcatcagtccgtac  catcatgggctccatcttcgccgctgtcttcgtcgccatctacaccaacaagcttccggg  ctacatggccagcattgtagtaccggcgctgcaaaagacatctctttctacagatgctat  tagtgaagtactagcgacagcacccactgcgaatcaggcagcactctcagctatcaaagg  cgtcactcctgagatcttgcgtgtcatcaacgccaaagtcgccgatgcttacggccactc  gtacgcctttgtttactacaccgctgctgctcttggggctgtatgcttctttgcggcaat  gtgtctgcgcgactttgatgtttatctgacagaccacgttgcccgaatg--  [25] FOTG_08309__Fusarium_oxysporum_f._sp._vasinfectum_25433  atgacgaaacaagaagcatcacaagatgacatctctccgggtcacaatgaccaggacgtt  gcactctcaaacgaagatgaacttcgttccaagatcttgactgctgagcgctcaactctc  ccagacggctacttcctcagccgcgagttcgttggaacattcggcggcattgctttcagt  cttgcagcgacatattttgccttccaggctggtgccggtgcactcatcaacatcaaccag  cacatcggccccagtgagaattactacctattctcaattgtgtggacggtctctcaaccc  atctcgatgctcttgtttggccgtctctctgacatgtttggtcgccgaaaccttgctctt  ggtgccaatgtcctcggtatctgtggtggtattattgctgccacttcccagagcatcaat  cagctcattgctgccaatgttctcttgggtcttgcctcgggcattccgggttcatatcct  cttttgacaggcgagcttatgacgaacaaactcaagtttcttggtactatcgttgttgta  gtgcccaatatcatcgccactggcttcggtccgtacctaggccttcgacttagcattctt  agttcatggagatggatcttttacatctacatcatcttaatgggtatgcctgcaaacttg  acacaacgcatggataactagaaccatgctgacaatatctcacagtaatcggtactaccc  tctggtacttcttctaccatcctccttcttttgtgcaacttcacggaacaagtatcagcc  gaaaggccgagcttgcaactatcgactgggttggcacttttcttcttgtctcgggccttg  ttctcttccttcttggaattgcctggggtgggcaaccgtacaa---atggacctcaggtc  gggtccttggtctcctgattcctggtgcagtttgctcgatcgcattcgttctttatgaga  tatatggccagcctgcgaagcctatcgtacccatgaaattctttaaggatgtccgaggtt  acacatgcgtcgtcatcatttccgccatcactggctgccttcaaacagcgctattcatat  tgtggccttcacaggttgcatacatctttggaagcaccaccaacggatgggaggagaccg  cttggatgtcaagtgttgtaaactttgcagcttgggccggtatcatcatcgtcgggcctc  tgtttcacgtcatcaagcatttgcgtctacagcttgttgtcggatctgcctggatgacag  ctttcctggcggccatgtctaccatcacctactccaacaagggttctgctattgcttttg  cctttctcagcacctttcccatcgggtggggagaagtcatgactatgctgatggttcagt  acattgttccggaacgagatctgggggttgcctttggtaagtaaccaaatctatcccgaa  ccctcctttcttcatgactaacagccttctttccaaagctgtcgtctcatcagtccgtac  catcatgggctccatcttcgccgctgtcttcgtcgccatctacaccaacaagcttccggg  ctacatggccagtattgtagtaccggcgctgcaaaagacatctctttctgcagatgctat  tagtgaagtactagcgacagcacccactgcgaatcaggcagcactctcagctatcaaagg  cgtcactcctgagatcttgcgtgtcatcaacgccaaagtcgccgatgcttacggccactc  gtacgcctttgtttactacaccgctgctgctcttggggctgtatgcttctttgcggcaat  gtgtctgcgcgactttgatgtttatctgacagaccacgttgcccgaatg--  [26] FOXB_15698__Fusarium_oxysporum_Fo5176  atgacgaaacaagaagcatcacaagatgacatctctccgggtcacaatgaccaggacgtt  gcactctcaaacgaagatgaacttcgttccaagatcttgactgctgagcgctcaactctc  ccagatggctacttcctcagccgcgagttcgttggaacatttggcggcattgctttcagt  cttgcagcgacatattttgccttccaggctggtgccggtgcactcatcaacatcaaccag  cacatcggccccagtgagaattactacctattctcaattgtgtggacggtctctcaaccc  atctcgatgctcttgtttggccgtctctctgacatgtttggtcgccgaaaccttgctctt  ggtgccaatgtcctcggtatctgtggtggtattgttgctgccacttcccagagcatcaac  cagctcattgctgccaatgttctcttgggtcttgcctcgggcattccgggttcatatcct  cttttgacaggcgagcttatgacgaacaaactcaagtttcttggtactatcgttgttgta  gtgcccaatatcatcgccactggcttcggtccgtacctaggccttcgacttagcattctt  agttcatggagatggatcttttacatctacatcatcttaatgggtatgcctgcaaacttg  acacaacgcaaggataactagaaccatgctgacaatatctcacagtaatcggtactaccc  tctggtacttcttctaccatcctccttcttttgtgcaacttcacggaacaagcatcagcc  gaaaggccgagcttgcaactatcgactgggttggcacttttcttcttgtctcgggccttg  ttcccttccttcttggaattgcctggggtgggcaaccgtacaa---atggacctcaggtc  gggtccttggtctcctgattcctggtgcagtttgctctatcgcattcgttctttatgaga  tatatggccagcctgcgaagcctatcgtacccatgaaattctttaaggatgtccgaggtt  acacatgcgtcgtcatcatttccgccatcactggctgccttcaaacagcgctattcatat  tgtggccttcacaggttgcatacatctttggaagcaccaccaacggatgggaggagaccg  cttggatgtcaagtgttgtaaactttgcagcttgggctggtatcatcatcgtcgggcctc  tgtttcacgtcatcaagcatttgcgtctacagcttgttgtcggatctgcctggatgacag  ctttcctggcggccatgtctaccatcacttactccaacaagggttctgctattgcttttg  cctttctcagcacctttcccatcgggtggggagaagtcatgactatgctgatggttcagt  acattgttccggaacgagatctgggggttgcctttggtaagtaaccaaatctatcccgaa  ccctcctttcttcatgactaacagtcttctttccaaagctgtcgtctcatcagtccgtac  catcatgggctccatcttcgccgctgtcttcgtcgccatctacaccaacaagcttccggg  ctacatggccagcattgtagtaccggcgctgcaaaagacatctctttctgcagatgctat  tagtgaagtactagcgacagcacccactgcgaatcaggcagcactctcagctatcaaagg  cgtcactcctgagatcttgcgtgtcatcaacgccaaagtcgccgatgcttacggccactc  gtatgcctttgtttactacaccgctgctgctctcggggctgtatgcttctttgcggcaat  gtgtctgcgcgactttgatgtttatctgacagaccacgttgcccgaatg--  [27] FsTRI12  atgac---------------------tgtcgtagttccag---aggaaggtctcgacctt  gaatctcagccggacgacaggatgagagccaaagccctcgccacttcagccgcagagcta  ccagacggatattacagatcacctcgcatcgtggcgtcctttgcagccttttcaatgaac  gtcgttgctacgtattttgttctccaagcatcagcttccgctcttcccaatatactccaa  gatgttggccagagtgaaaactcaagtctcttctcgactctatggacgactggccaagct  gtcagtattctgatgatgggtcgcctcacggacagatttgggcgacggccatttgttatt  cttactcatattctgggactcgtcggcgctatcgttggatgcacagctaccaaattcaat  actctcttggctgcaatgacgatgctgggtgttgccgctggcccagcaggtgccagtcct  ctattcattggcgagctgatgagcaacaaaaccaagtttttgggtcttctcatcgtatct  gctcccgttgtcgccacgaatggtcttagcccctaccttggtcagcgtcttgctatacag  ggcagttggcgttggatcttctacatttatatcataatgagtagtaag-ttgtgtgtttc  ttgcatcgaaggcacggt---aacgatgctgacat--cttgacagcaattgcagtcacac  ttatcattatctggtactaccccccgtcatttgcgcaacttcacgggaaaaaggtcagca  agagagaggaactagcaaaagtcgattggataggtatcatacttgttattgccggaacat  cgctctttcttcttggcgtttcttggggtgggcagccgaacaacccgtggaactctgcca  aggtcatcggactcatatcatccggcgctggcactctcgttatctttgccttgtacgagg  tctatggcaagcccgaacgccccatggttccgcctagtctcttcaaagatactcgtggct  ttgtctgcattcttatcatcagctccatcatgggctcgatgcacctttcccttgttatca  tgtaccctcagcaagttgtcaatatcttcggctctagtttgaagaactgggaagagactg  catggatgtcagcgactgcctcattcggcacaggtgctggagtggtggttcttggtagct  tgtttcatcttgtcaggcacatccgttggcaaatacttgtcggagctatgtggcttactg  ctttcctcggagccatgtcatctatcaaccgagacaacaagaactctgccattgctttgt  cagttatgaccggctttgtcgtcgcctgggctcaggacatcactatgctcctagtccaat  tcatcacgacagatgaaaacttaggcgtggcctttggtatgtagtcctatctatactga-  -----------tgataccttactaagatttcgt-atagctgttgttgctgcggcccgccc  ctttgccggctctatcttcaccgccgccttcatctccgtctacaccaaccggtatccgcg  cgagctggcaacccatcttagttcagccctgcgtggtacgggctttccacaaggaagttt  ctccagtcttcttgaggccgccaagtcgggacgaatggaagccgtgaacgctctccctgg  gatgacgaccgaaatctcttcggtggtcagccaagctatggcggatagttacacagcttc  atatgctaatgtctattacttcgccatggctctgggtgtcattccaatcattgccagcct  ttgtatgagggatttggactgctacttaactgaccacgttccgcatcag--  ;  END;  BEGIN SETS;  CHARSET 'exon1' = 1-643;  CHARSET 'intron1' = 644-705;  CHARSET 'exon2' = 706-1416;  CHARSET 'intron2' = 1417-1493;  CHARSET 'exon3' = 1494-1849;  END; |
| --- |
